# Macroporous chitosan/alginate hydrogels crosslinked with genipin accumulate and retain glioblastoma cancer cells†

**DOI:** 10.1039/d4ra06197g

**Published:** 2024-11-05

**Authors:** Lauriane Parès, Sahar Naasri, Lisa Delattre, Hélène Therriault, Benoît Liberelle, Gregory De Crescenzo, Marc-Antoine Lauzon, Nathalie Faucheux, Benoit Paquette, Nick Virgilio

**Affiliations:** a Research Center for High Performance Polymer and Composite Systems (CREPEC), Department of Chemical Engineering, Polytechnique Montréal Montréal H3C 3A7 Québec Canada nick.virgilio@polymtl.ca; b Center for Research in Radiotherapy, Department of Nuclear Medicine and Radiobiology, Faculty of Medicine and Health Sciences, Université de Sherbrooke Sherbrooke J1H 5N4 Québec Canada benoit.paquette@usherbrooke.ca; c Department of Chemical Engineering, Polytechnique Montréal Montréal H3C 3A7 Québec Canada; d Department of Chemical and Biotechnological Engineering, Faculty of Engineering, Université de Sherbrooke Sherbrooke J1K 2R1 Québec Canada

## Abstract

Grade IV multiforme glioblastoma (GBM) is an aggressive cancer that remains incurable due to the GBM cells invading and proliferating in the surrounding healthy tissues, even after tumor resection. A new therapeutic paradigm to treat GBM is to attract and accumulate GBM cells in a macroporous hydrogel inserted in the surgical cavity after tumor resection, followed by a targeted high dose of radiotherapy. This work presents a molding-based method to prepare macroporous hydrogels composed of sodium alginate and chitosan, homogeneously mixed in solution using sodium bicarbonate, and subsequently crosslinked with genipin and calcium chloride. The gels display a blue color, the result of chitosan crosslinking with genipin, fully interconnected pores with an average diameter of 180 μm (and tunable over a wide range), with a compression modulus of 10 kPa, close to the value of brain tissues. The gels are stable in cell culture media and keep their integrity after radiation doses comparable to current GBM treatment levels. Finally, F98 GBM cells accumulate relatively homogeneously and are retained within the gels.

## Abstract 2

This work demonstrates how macroporous hydrogels can be prepared with porous polylactide molds, and homogeneous solutions of sodium alginate and chitosan crosslinked with genipin, in order to accumulate glioblastoma cancer cells.

## Graphical abstract

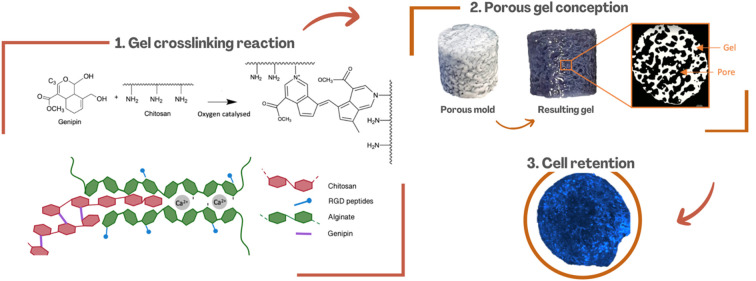

## 1 Introduction

Grade IV multiforme glioblastoma (GBM) is the most aggressive type of cancer located in the brain or spinal cord, with a survival rate of 5%, 5 years after diagnosis.^1^ Current treatments combine surgery, radiotherapy, and chemotherapy. Surgery may improve the patients' quality of life in the short term by removing the pressure caused by the growing tumor volume, but it has to be combined with radiotherapy and chemotherapy to eradicate the remaining GBM cells. However, GBM cells also invade the surrounding healthy brain tissues where they proliferate.^2^ Unfortunately, these healthy tissues are more sensitive to the current treatments than GBM cells, which limits the doses of radiation and chemotherapeutic agents that can be delivered.^3^ Consequently, and despite these treatments, cancer recurrence is currently inevitable.

A number of new biopolymer-based therapies are being developed to treat GBM. The high heterogeneity of GBM tumors suggests that the ideal therapy will likely be a patient-and-tumor-specific combination of drugs delivered at optimal release rates, which will then require different polymers and formulations for each drug.^4^ One such approach, the GLIADEL wafer, is based on an interstitial drug delivery and has been approved for clinical use. Following tumor resection, the wafers are placed in the surgical cavity. This bio-device gradually releases carmustine directly to the tumor area, allowing for prolonged, targeted exposure of the residual cancer cells to this chemotherapeutic agent. However, benefits are still modest. The administration of non-selective drugs for GBM cells leads to mixed clinical results and various complications arise.^5,6^ The maximal tolerated dose by the patient does not frequently reach significant tumoricidal activity for the GBM cells, in addition to the rapid clearance of the drugs from the brain.

Another modality proposed recently is to remove the GBM cells that are infiltrated in the brain tissues, by attracting and concentrating those inside an hydrogel inserted in the surgical cavity – *i.e.* a cancer cell trap. GBM cells will be attracted by chemoattractants released by the hydrogel, in which they will accumulate *via* a network of pores. Once into the trap, the next step will be to perform stereotaxic radiotherapy, a localized irradiation technique, at a high dose, to eradicate the GBM cells. A larger number of GBM cells will be eliminated without delivering a high and toxic radiation dose to the healthy brain tissues, which will improve the prognosis and the patient's quality of life.

Various hydrogel materials and chemoattractants have been recently tested. Najberg, Haji Mansor, Boury, Alvarez-Lorenzo & Garcion (2019)^7^ have used tissue engineering techniques to construct an artificial environment that replicates tumor characteristics by designing a unique three-dimensional matrix incorporating molecular signals that attract tumor cells and effectively trap them. Autier *et al.* (2019)^8^ have developed a bacterial cellulose implant loaded with human serum albumin (HAS), whereas a recent study evaluated the capacity of the chemoattractant CXCL12 to attract residual glioblastoma stem cells into a thiol-Michael hydrogel, confirming the specific attraction of GBM cells into the gel.^9^

Recently, we reported the development of a sodium alginate/chitosan (SA/CHI) based macroporous hydrogel for the capture and accumulation of GBM cells.^10,11^ The fabrication technique employs porous polymer molds obtained from melt-processed co-continuous polymer blends, allowing fine control over the porosity features of the hydrogels, including an average pore size tunable from ≈5 μm to 500 μm, and with fully interconnected pores.^12^ The method is also compatible with a variety of gel chemistries.^13^ SA is derived from brown algae and comprises a family of anionic linear random block copolymers (p*K*_a_ = 3.5) composed of mannuronic (M) and guluronic (G) residues (Fig. S1†). It has gained strong interest in the biomedical field because of its biocompatibility, non-immunogenicity, abundance, and low cost. SA gels display mechanical properties close to certain soft tissues of the human body.^14^ It is used in wound healing, drug delivery, *in vitro* cell culture and tissue engineering applications.^14^ SA gels are typically prepared by ionic crosslinking with divalent cations in solution (usually Ca^2+^), following the “egg-box” model.^14^ However, SA gels can lack stability in physiological media because of ionic exchanges (with Na^+^ for example) with the surroundings.

Chitin is a component of the exoskeleton of arthropods and is also found in the cell walls of fungi and yeasts. It is a nitrogenous polysaccharide obtained from the polymerization of *N*-acetylglucosamine units bound together by an osidic bond. Chitosan is then obtained by the deacetylation of chitin, typically above a degree of 50% (Fig. S1†). It is a semi-crystalline polysaccharide, soluble in acidic aqueous solutions (p*K*_a_ = 6.3) due to the protonation of the amine groups. Its solubility also depends on its deacetylation degree, the ionic concentration, solution pH and nature of the acid used, and finally the distribution of the acetyl groups along the chain, since they participate in inter-chain hydrophobic interactions.^15^

**Fig. 1:**
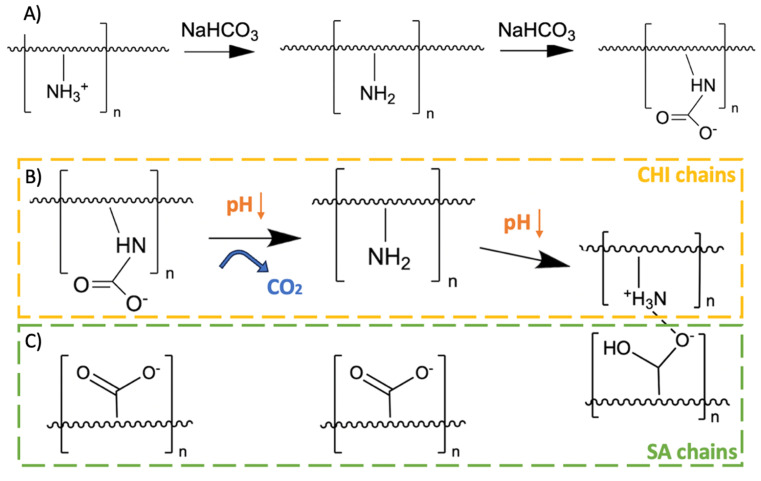
(A) Chitosan (CHI) reaction with sodium bicarbonate, (B) regeneration of primary amines when the pH decreases again, and (C) interactions with sodium alginate (SA) chains once CHI recovers its amino groups.^16^

While a direct combination of SA and CHI in hydrogels provides additional stability in physiological medium due to their complementary charges,^11^ two issues remain: (1) directly mixing SA and CHI results in the formation of aggregates (heterogeneities) in the gels due to their strong electrostatic interactions, and (2) dissolution in physiological media is only delayed, but not stopped. Komoto, Furuike & Tamura (2019) have shown that adding sodium bicarbonate in excess to a CHI solution renders CHI soluble in basic medium.^16^ Indeed, carbonate and bicarbonate ions react with the CHI amino groups to form anionic carbamate groups, while increasing the solution pH (Fig. 1A). Mixing CHI with SA in solution then results is a fully homogeneous and transparent solution devoid of aggregates. This reaction is reversible by decreasing the pH again. Indeed, the C–N bonds are cleaved and CO_2_ is released (Fig. 1B). When CHI gradually becomes cationic again, attractive electrostatic interactions between SA and CHI chains develop – only in that case more slowly, in a controllable way, yielding homogeneous gels (Fig. 1C).

**Fig. 2:**
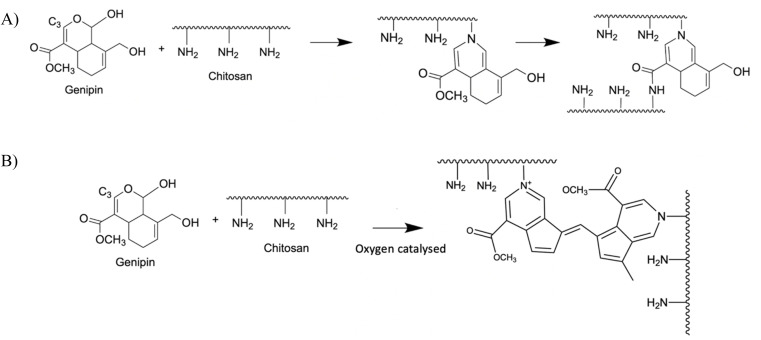
Crosslinking reaction of CHI chains by GNP in acidic (A) or alkaline (B) media.^18^

In addition, chitosan can also be chemically crosslinked with genipin (GNP). Genipin is an iridoid, *i.e.* a secondary plant metabolite, produced by *Gardenia jasminoides* and *Genipa americana* L as a mean of defense against microbial infections. It displays a variety of medicinal effects, including anti-inflammatory, anticancer, antithrombotic and antibacterial properties.^17^ GNP reacts spontaneously with the primary amines of amino acids, proteins and peptides and forms bluish pigments. It has been used as a crosslinker for chitosan by reacting with its amino groups, forming heterocyclic amines as bridges between chitosan chains.^18^ There are two crosslinking mechanisms, depending on the pH of the solution. In acidic and neutral pH, there is a first nucleophilic attack of the C3 carbon of GNP on the primary amine groups of chitosan, leading to the formation of a heterocyclic compound linked to the glucosamine residue of chitosan (Fig. 2A). This is followed by the nucleophilic substitution of the ester group present in GNP, releasing methanol and forming a secondary amide bond with chitosan. Under alkaline conditions, there is a nucleophilic attack by hydroxyl ions in aqueous solution (Fig. 2B) forming intermediate aldehyde groups, which then undergo crotonization (a crotonate group –CH=CH–CH_3_ is formed into the molecule).^17^ The terminal aldehyde groups of the polymerized GNP then undergo a Schiff reaction with the amine groups of chitosan to form cross-linked networks.

The research hypothesis and main objective of this work was to enhance the stability of SA/CHI macroporous hydrogels in physiological media for the capture of GBM cells, by combining the protocols developed by Komoto, Furuike & Tamura (2019) for the preparation of homogeneous SA/CHI gels, with the chemical crosslinking of CHI with GNP.^16^ Macroporous gels with a compression modulus ranging from 1 to 10 kPa, comparable to the properties of brain tissues, were targeted. The gels were prepared following the technique developed in ref. 12. The gelation kinetics were monitored as a function of SA, CHI and GNP compositions, as well as temperature, by rheometry and UV-vis spectroscopy. The resulting mechanical properties, stability in physiological media, and stability as a function of irradiation dose, were evaluated by measuring and monitoring in time the unconstrained compressive mechanical properties. Finally, the capacity to accumulate and retain GBM cells within the hydrogels was evaluated.

## 2. Materials and method

### 2.1 Materials

Sodium alginate (SA) IL-6G grade was purchased from Kimica (Pain, Chile) with the following characteristics: mannuronate/guluronate (M/G) ratio = 0.5, viscosity: 30–60 mPa s, molecular weight Mn = 67 ± 8 kDa.^11^ Chitosan (CHI) 85/60/A1 grade was obtained from BioLog Heppe GmbH, Landsberg, Germany: degree of deacetylation (DDA) = 85%, viscosity: 60 mPa s, *M*_n_ = 50–100 kDa (from supplier). Polylactic acid Ingeo 4032D (PLA, melt flow index = 7 g/10 min@210 °C/2.16 kg, *ρ*(200 °C) = 1.11 g cm^−3^, melting temperature *T*_m_ = 169 °C, from supplier) was purchased from NatureWorks, and polystyrene MC3650 (PS, melt flow index = 13.0 g/10 min@200 °C/5 kg, *ρ*(200 °C) = 0.97 g cm^−3^, glass transition temperature *T*_g_ = 94 °C, from supplier) was obtained from Americas Styrenics. Cyclohexane, chloroform, sodium hydroxide (NaOH), acetic acid, and hydrochloric acid (HCl) were all ACS-grade chemical reagents. Calcium chloride (CaCl_2_), sodium bicarbonate, *N*-hydroxysuccinimide (NHS, 98% purity), ethyl-*N*′-(3-dimethylamino-propyl) carbodiimide hydrochloride (EDC, 99+% purity), phosphate buffered saline (PBS, pH = 7.4, powder form) and sodium azide S2002 (≥99.5%) were purchased from Sigma-Aldrich. *N*-ε-Maleimidocaproic acid hydrazide (EMCH), Dulbecco's Modified Eagle Medium powder (DMEM) and genipin (GNP) (*M*_w_ = 226.23 g mol^−1^, purity > 98%) were purchased from Thermo Fisher Scientific. The cysteine-terminated peptide CGGRGDS (RGD) was supplied by EZBiolab (98%, *N*-terminal acetyl, C-terminal amide, New Jersey, USA). All antibiotics, penicillin, streptomycin and amphotericin B were purchased from Sigma-Aldrich. All aqueous solutions were prepared with Milli-Q water (18.2 MΩ cm, total organic compounds ≤5 ppb). The glassware was carefully cleansed by overnight immersion in a bath of KOH-saturated isopropyl alcohol, followed by intensive rinsing with Milli-Q water, and tools were cleaned with Milli-Q water and ethanol 70% v/v before use.

### 2.2 Preparation of PLA macroporous molds

Blends of PS and PLA (50/50 wt%) were melt-processed and pelletized using an AG 34 mm Leistritz co-rotating twin-screw extruder at 100 rpm and 190 °C with one feeder and equipped with a post-processing water cooling bath and a pelletizer. This ratio was chosen to obtain a co-continuous morphology.^19^ The pellets were next melt-extruded and shaped into rods (2 cm diameter) at 16 rpm in a single screw extruder (Killion KN-150) and quenched into water maintained at approximately 45 °C to freeze-in the desired morphology. The rods were next cut into 12 cm bars and annealed using a hot press at 190 °C for 5, 10, 20, 30, 45 and 60 min to allow microstructure coarsening, before quenching again in water. The annealed bars were trimmed with a numerically controlled instrument (CNC, Genmitsu 3020-PRO MAX Router) to obtain small cylinders (diameter *ϕ* = 6.5 mm, height *h* = 6 mm). Finally, the PS phase was extracted in a soxhlet apparatus using cyclohexane for one week. The resulting porous cylinders were dried overnight in a vacuum oven at 40 °C.

### 2.3 Preparation of alginate, chitosan and genipin stock solutions

#### 2.3.1 Filtration and freeze-drying

SA powder was dissolved in hot water at 1% w/v and sonicated until complete dissolution. The solution was filtered next with PROGENE vacuum filtration units (polyether sulfone membrane, 0.22 μm pore size, Ultident Scientific, Saint-Laurent, Quebec, Canada) and freeze-dried. CHI flakes were dissolved at 1% w/v in a 0.1 M HCl solution and sonicated until complete dissolution. The solution was filtered likewise and brought to a neutral pH using a 5 M NaOH solution before freeze-drying.

#### 2.3.2 RGD grafting on SA

Freeze-dried SA was dissolved in 40 mL of Milli-Q water at 1% w/v. 3.5 μL of 0.1 M NHS and 1 μL of 0.4 M EDC were added to the solution and left on a shaker plate for 20 min at room temperature (RT). 17.5 μL of a 10 mM aliquot of EMCH dissolved in DMSO was then added and the solution was left on the shaker plate for 60 min. Finally, 56.9 μL of a 2 mg mL^−1^ RGD solution was added to the solution, for a total of 3.5 × 10^−7^ mol of cysteine-terminated peptide per 400 mg of SA. The solution was maintained under agitation for at least 20 h and filtered next five times to withdraw the remaining free peptides and reagents using Amicon Ultra-15 10 kDa membrane centrifugal filter units (Merck Millipore, Tullagreen, Ireland). The resulting RGD-grafted SA solution was freeze-dried and stored at 4 °C until use.

#### 2.3.3 SA, CHI and GNP stock solutions preparation

Freeze-dried SA was dissolved in hot milliQ water at 2% and 2.5% w/v and sonicated in a warm bath until complete dissolution. Freeze-dried CHI was dissolved in a 0.1 M acetic acid solution at 0.5%, 0.75%, 1%, 1.5% and 2% w/v, and sonicated until complete dissolution. Sodium bicarbonate powder was then added at 4.5% w/v, followed by vigorous stirring, and degassing under the hood. The resulting solutions were completely transparent, and the pH was in between 7 and 8. GNP powder was dissolved at 0.3% w/v in milliQ water and sonicated until complete dissolution. All solutions were stored at 4 °C until use.

#### 2.3.4 SA/CHI/GNP mixed solutions preparation

Various gelling formulations were prepared. SA/CHI solutions were prepared by mixing together the required volumes in order to obtain the following ratios: 0.5/0.5, 1/0.5, 1/0.75 and 1/1% w/v. A volume of GNP stock solution was then added in order to obtain a concentration range of 0.0125 to 0.075% w/v based on the CHI composition (Tables S1 and S2†). For example, for a final CHI concentration of 0.75% w/v in solution, a GNP composition of 0.0125% w/v means a CHI/GNP weight ratio of 60 in the final solution.

### 2.4 Preparation of SA/CHI/GNP macroporous hydrogels

#### 2.4.1 Injection of gelling solution in the polymer molds and gelation

Porous PLA molds were placed into 50 mL falcon tubes, and the tubes were next filled with a SA/CHI/GNP solution. A small grid was placed on top of the molds to maintain them fully immersed. The lidless falcons were deposited in a custom-built injection system and 3 to 4 vacuum – nitrogen (N_2_) pressure cycles were applied to fill the molds. Once filled, the molds were gently wiped on their surface to remove the excess of solution, and they were placed in closed Eppendorf tubes at 37 °C in an oven for 24 h to crosslink the CHI with GNP. The filled molds were finally plunged into a 0.22 μm filtered 4% w/v CaCl_2_ solution at 4 °C overnight to initiate SA gelation.

#### 2.4.2 PLA molds dissolution

Once the gelation process completed, the PLA molds filled with the gels were placed into vials containing about 10 mL of chloroform and were gently stirred on a shaker plate at 60 rpm to selectively dissolve the PLA phase. The chloroform was changed every two days until the PLA phase was completely dissolved, which corresponded to 3 or 4 solvent changes. Macroporous gels were then rinsed twice with MilliQ water and stirred on a shaker plate for at least one day between the two cleanings, and finally stored in a 4% w/v CaCl_2_ solution until use.

### 2.5 Gelation kinetics monitoring

#### 2.5.1 UV-vis spectroscopy

0.5 mL of CHI/GNP solutions (CHI range: 0.25 to 1% w/v; GNP: 0.0125 to 0.075% w/v) were poured into a 48-well plate and were left to gelify at RT, 37 °C or 50 °C (*N* = 3 per solution composition). Half of the wells were filled with the gelling solutions and the other half with water to prevent the gels from drying out. The absorbance was monitored by UV-vis spectroscopy with a Victor V Multilabel Counter from PerkinElmer Inc. (Woodbridge, ON) equipped with a plate reader, as a function of time, from 300 nm to 800 nm to identify the maximum absorbance peak, then at 595 nm (based on Fig. S2†) over a week for each composition. Absorbance values were finally normalized, first by subtracting the value of the CHI solution without GNP, then by dividing the results by the maximum value obtained for a given set of experiment and multiplied by 100 – setting the maximum value at 100%.

#### 2.5.2 Rheometry

The gelling kinetics of CHI/GNP and SA/CHI/GNP solutions were also monitored with a stress-controlled Anton-Paar Physica MCR 501 commercial rheometer with a cylindrical geometry (bob diameter: 16.66 mm; cup diameter: 18.066 mm).^20^ Dynamic time sweep measurements were performed in the LVE regime at a strain amplitude *γ*_0_ = 3% and an angular frequency *ω* = 1 rad s^−1^ for 48 h. Solutions in the geometry were covered with a thin film of low viscosity mineral oil to prevent drying. The storage (*G*′) and loss (*G*′′) moduli were plotted as a function of time to determine the gelation time *t*_gel_, chosen here when Tan *δ* (=*G*′′/*G*′) = 1,^21^ and the equilibrium values once a plateau was reached at gelation completion.

### 2.6 Characterization of PLA molds and SA–CHI hydrogels

#### 2.6.1 X-ray microtomography and porosity quantification by image analysis

Porous PLA molds were analyzed by X-ray microcomputed tomography (microCT) using a Zeiss Xradia 520 Versa instrument with the following parameters: 40 keV (source voltage), 250 μA (source current), 9.80 μm (image voxel size), 16 bits (depth), 0.4° (rotation step) and an exposure time of 1 s and 0.4 or 4× objectives according to the pore size. The structure of the PLA molds was then visualized by reconstructing the 3D structure using the Dragonfly software. The microstructure of a SA/CHI/GNP macroporous gel (for an annealing time of 30 min) has also been characterized by removing first the free water contained in the pores with absorbent paper in order to provide contrast between the gel phase and the pores.

After data acquisition and 3D reconstruction, image analysis was realized by applying a Noise Reduction Filter (NRF) with the Scout-and-scan Control System Reconstructor. The data were then post-processed with the Dragonfly software. The contrast was first modulated to obtain a black and white reconstruction, with the pores in black and the PLA (or gel) phase in white. Each domain (PLA, gel, or pores) was then selected in order to create masks. A cylindrical cut was then realized to remove the sample holder and outside environment. The thickness mesh of each domain type (PLA, PS, gel and pores) was obtained using a built-in algorithm, giving the average domain sizes, size distributions and interfacial area/pore surface. The specific interfacial area/pore surface was finally obtained by dividing by the sample volume.

#### 2.6.2 Compression moduli of macroporous hydrogels

Compression tests were performed with a Mach-1 mechanical tester (Biomomentum, Laval, Canada, Actuator A400.25, load cell of 150 g, 10 mm diameter indenter) on bulk and porous gels. The indenter diameter was chosen to be slightly larger than the diameter of the gels. In addition to the porous gels, bulk gels (without pores) were cut with a 5 mm diameter biopsy punch. All gels were fully covered with water in the instrument testing cell to avoid the effects of capillary forces. Unconstrained compression tests were carried out in two steps: (1) the indenter established contact with the gel (speed = 25 μm s^−1^, stop criteria: 0.25 g), at the end of which the testing cell reservoir was filled with water; (2) a first pre-compression (displacement of 50 μm) at the same speed was applied to make sure that the probe was fully in contact with the gel surface, followed by two compressions, each totaling a 25 μm displacement at 20 μm s^−1^ on the gels. The compression modulus was calculated as follow: the Mach-1 software records the displacement Δ*h* in μm and the applied load *G* in kg at an acquisition rate of 0.1 s^−1^. By measuring the dimensions of the gels (diameter *d* and height *h*_0_) with an electronic micrometer prior to testing, and by assuming that the diameter remains constant at such small strains, the stress (*σ*)/strain (*ε*) curve is obtained following eqn (1) and (2):1
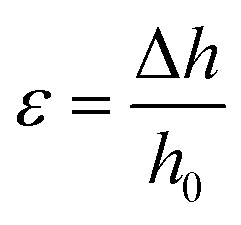
2
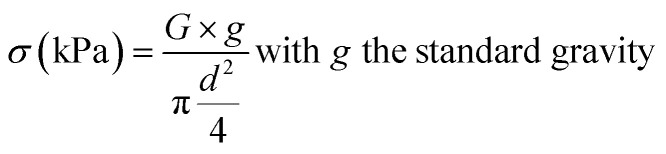


Linear regressions of the data (slope) obtained for the last two steps in compression yielded the compression modulus. The values are reported as the average ± one standard deviation, with a total of 5 gels per formulation (*N* = 5).

#### 2.6.3 Dimensional and structural stability tests in saline and cell culture media

The stability of the porous gels has been assessed during two weeks by monitoring their dimensions and compression modulus in four different media. First, two solutions of antibiotics were prepared: (1) a solution of penicillin G 6.1% w/v and streptomycin 10% w/v has been prepared with Milli-Q water and then filtered at 0.22 μm and stored at −20 °C until use; (2) In the same way, an amphotericin B solution at 1.25% w/v was prepared. Next, 100 μL of these two solutions were added to 500 mL of three (out of four) tested media, which consisted in: (a) a 4% w/v CaCl_2_ control solution prepared with Milli-Q water and then filtered at 0.22 μm without any antibiotics; (b) a 4% w/v CaCl_2_ control solution supplemented with antibiotics; (c) a Dulbecco's Minimal Essential Medium (DMEM) with antibiotics, and (d) a PBS 1× solution with antibiotics.

The porous gels were first sterilized in 70% v/v aqueous ethanol for 10 min on a shaker plate. Next, they were washed three times with Milli-Q water during 20 min and they were left overnight in a 4% w/v CaCl_2_ solution. The day after, they were first rinsed with their assigned media, and they were deposited next in 2 mL of the same media in a 5 mL Eppendorf, with a total of 3 gels per medium. Gel stability was assessed by monitoring their dimensions with a caliper, and by measuring their compression modulus with the Mach-1 tester, as described previously. Half of the gels in CaCl_2_ media were not mechanically tested as controls. Each solution was changed every 3 days, except for 3 additional gels into the PBS media to assess the impact of solution changing on the gel properties.

#### 2.6.4 Structural stability of hydrogels after irradiation

The compression modulus of macroporous hydrogels (SA 1% CHI 0.75% GNP 0.05% w/v, and average pore size of 180 μm) were first measured as described previously in Section 2.6.2. They were next stored in a CaCl_2_ 4% w/v solution supplemented with sodium azide (6 droplets of sodium azide 5% w/v in 500 mL of CaCl_2_ 4% w/v). Five days later, the gels were rinsed twice with distilled water. The gels were then placed in a Petri dish and were irradiated with doses of 10, 15, 20 or 30 Gy (*N* = 4 gels per irradiation dose). The dose was delivered by a cesium-137 fixed source (Gammacell 3000 Elan, Best Theratronics, Ottawa, Canada). One week later, compression tests were repeated on gels irradiated at a dose of 30 Gy.

### 2.7 Cell culture

The F98 murine GBM cell line was purchased from American Type Culture Collection (Manassas, VA) and was cultured in a DMEM medium supplemented with 10% fetal bovine serum, a mixture of antibiotics (100 U per mL of penicillin and 100 μg mL^−1^ of streptomycin), 44 mM sodium bicarbonate and 2 mM l-glutamine. Then the cells were placed in a humidified incubator with 5% CO_2_ at 37 °C.

In order to introduce the mCherry marker into F98 cells, the pCDH-CMV-mCherry-T2A-Puro lentiviral vector (Addgene Catalog#72264, Watertown, MA) was used.^22^ Cells expressing the mCherry marker were incubated for 10 days in DMEM containing 2.5 μg mL^−1^ of puromycin. The expression of mCherry by F98 cells was confirmed by observation with a Leica DM-IRBE fluorescence microscope.

#### 2.7.1 Sterilization of hydrogels

One day before use, the hydrogels were incubated in the presence of 70% ethanol in a 24-well plate for 10 min, then rinsed with distilled water for 20 min, after which they were kept overnight at 4 °C in a solution of 4% w/v CaCl_2_. The next day, the gels were rinsed twice for 20 min in Dulbecco's Minimal Essential Medium (DMEM) without any additives, then stored in DMEM + BSA medium at 4 °C.

#### 2.7.2 Measurement of F98 mCherry cells fluorescence intensity in presence of hydrogels

The fluorescence of F98 mCherry cells accumulated in SA 1% CHI 0.75% GNP 0.05% w/v hydrogels was determined and compared to those formulated with either SA 1% w/v, or with SA 1% CHI 0.75% w/v. All hydrogels were grafted with the RGD cell adhesion peptide. Sterilized hydrogels, 5 mm in diameter and 4 mm high, were carefully placed in a 96-well plate, then different concentrations of F98 mCherry cells (0; 0.1 × 10^6^; 0.5 × 10^6^; and 1 × 10^6^) in 100 μL of DMEM were added dropwise onto the hydrogels surfaces. After 24 h of incubation at 37 °C and 5% CO_2_, the hydrogels were placed in a new 96-well plate containing 100 μL additive-free DMEM and fluorescence reading with a microplate reader (*λ*_ex_ = 530 nm, *λ*_em_ = 590 nm, HT Synergy, Bio-Tek Instrument, Winooski, VT, USA) was performed.

#### 2.7.3 Accumulation and retention of F98 cells in the hydrogels: Alamar Blue assay

As negative control, sterile hydrogels were placed in a 24-well plate and 700 μL of DMEM media containing 10% Alamar Blue was gently added. After 2 h at 37 °C and 5% CO_2_, 100 μL of this media was taken and transferred to a 96-well black opaque plate (Corning incorporated, Catalog#3915, NY, USA) to perform fluorescence readings (*λ*_ex_ = 530 nm, *λ*_em_ = 590 nm).

The hydrogels were then placed in a 96-well plate and F98 cells without mCherry (10^6^ in 100 μL of DMEM per well) were gently added onto the hydrogel surface. After 24 h of incubation, the hydrogels were gently transferred in a 24-well plate containing DMEM media and 10% Alamar Blue, and incubated for 2 h at 37 °C. The number of F98 cells accumulated in the hydrogels was determined by measuring the fluorescence of oxidized Alamar Blue. The hydrogels were then transferred in a migration chamber (TC-Insert, Sarstedt, Catalog#83.3922.800, Nümbrecht, Germany) connected to a 20 mL syringe to carry out stringent washing, and then transferred to a 24-well plate containing 700 μL of DMEM media and 10% Alamar Blue to determine the number of F98 WT cells retained in the hydrogels.

#### 2.7.4 Distribution of F98 cells within the hydrogels

F98 cells (0.5 × 10^6^) were added gently on the top of the hydrogels. After incubation for 24 h at 37 °C and 5% CO_2_, the cells were fixed with 3% w/v paraformaldehyde (PFA) and then stained with rhodamine–phalloidin 2 105 247 (actin cytoskeleton, red channel) (1 : 100) and Hoechst 33 342 (nuclei, blue channel) (ThermoFisher Canada), at a final concentration of 5 μg mL^−1^ in 0.1% w/v BSA in DMEM red phenol-free media.

The distribution of F98 cells was evaluated at 4 different levels (L) – the first level (L1) was located 1 mm deep from the upper surface of the hydrogels, while the last level (L4) was 3.5 mm deep. F98 cell distribution within the hydrogels was observed using an epifluorescence microscope (EVOS FL Auto Imaging System, Life Technologies). The images were then analyzed with tools developed by our team in order to determine the number and the size of the clusters of F98 cells in each level.^10^ Briefly, for a given image, the program selects cell clusters that are in the focal plane, and leaves out the others, before counting and measuring.

### 2.8 Statistical analysis

Results are expressed as the mean ± standard deviation of 2 to 4 experiments performed in triplicate. Statistical analyses were performed using two ways analysis of variance (ANOVA). A value of *p* < 0.05 was considered to be statistically significant. *p < 0.05, **p < 0.01, ***p < 0.001, ****p < 0.0001.

## 3. Results

### 3.1 Kinetics of CHI crosslinking reaction with GNP

#### 3.1.1 UV-vis spectroscopy

**Fig. 3:**
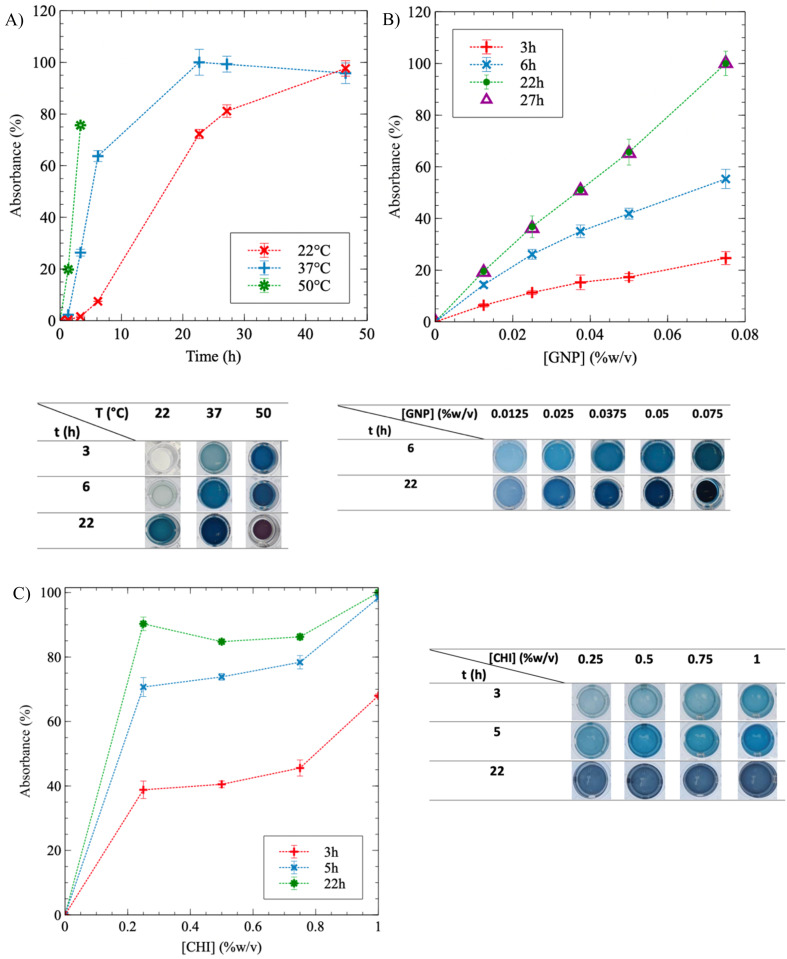
Chitosan (CHI) crosslinking kinetics using genipin (GNP) monitored by UV-vis spectroscopy. (A) Absorbance as a function of time *t* and temperature *T*, at [CHI] = 0.75% w/v and [GNP] = 0.05% w/v; (B) absorbance as a function of GNP composition and reaction time, at [CHI] = 0.75% w/v and 37 °C; (C) absorbance as function of CHI concentration at [GNP] = 0.025% w/v and *T* = 37 °C. Three samples were tested per condition (*N* = 3), the dotted lines are guides for the eyes. After 22 h, the absorbance remained constant in all cases (data at longer times are not shown). Absorbance values (%) are normalized using the maximum value obtained for a given experiment. Pictures of well-plates containing CHI hydrogels at various conditions are displayed with the graphics.

GNP, used to crosslink CHI, produces blue pigments during the reaction, allowing the reaction progress to be monitored by UV-vis spectroscopy (see photos of the well-plates containing the gels in Fig. 3). The color intensity increases as crosslinking progresses. The full absorbance spectra for 0.75% w/v CHI hydrogels were obtained, and a peak at 595 nm was identified (Fig. S2†). Hence, only the absorbance value at 595 nm was considered for subsequent analyses.

To investigate the effect of temperature on CHI gelation, the crosslinking kinetics were monitored at three different temperatures: 22 °C (room temperature), 37 °C and 50 °C. When [CHI] = 0.75% and [GNP] = 0.05% w/v, Fig. 3A demonstrates that as *T* increases, the reaction accelerates (the curves shift to the left), and it reaches completion – *i.e.* a plateau at 100% – more rapidly. After 3 h, a noticeable difference in color is observed: the solution remains transparent at 22 °C, becomes a clear blue at 37 °C, and dark blue at 50 °C. At the end of the experiment, similar plateau values were obtained for reactions carried out at 22 °C and 37 °C, but the reaction time at 22 °C was twice as long compared to 37 °C – 46 h and 22 h, respectively. Gelation at 50 °C resulted in syneresis, with the gel shrinking and surrounded by water after 6 h, as the pictures of the gels in well-plates below the graphic in Fig. 3A show. Syneresis was also observed at 22 °C for prolonged gelation times exceeding 48 h (not shown). Thus, a temperature of 37 °C provides a favorable compromise, reducing gelation time while minimizing syneresis. The same approach was employed to quantify the influence of GNP concentration on CHI gelation (Fig. 3B). During the crosslinking reaction, absorbance exhibited a quasi-linear relationship with GNP content, and the color gradually darkened accordingly. A higher GNP concentration resulted in more extensive crosslinking during and at the end of the experiment. However, an excessive GNP composition led to syneresis, as observed at a GNP concentration of 0.075% w/v after 22 h of crosslinking. Consequently, the GNP concentration was limited to 0.05% w/v in the GNP/CHI solutions. Similar trends were obtained at lower CHI and GNP concentrations (Fig. S3†). Modulating the CHI composition resulted only in a slight difference in absorbance between the solutions, at a given reaction time, as the GNP content remained constant (Fig. 3C). The slightly higher absorbance observed at 1% w/v of CHI is likely due to the increased availability of CHI chains. At reaction completion, the gels reached approximately the same absorbance values and exhibited similar blue colors. This suggests that GNP is likely the limiting reactant and was not added in excess. In addition, solutions at higher CHI concentrations were not fully crosslinked at similar GNP concentrations.

**Fig. 4:**
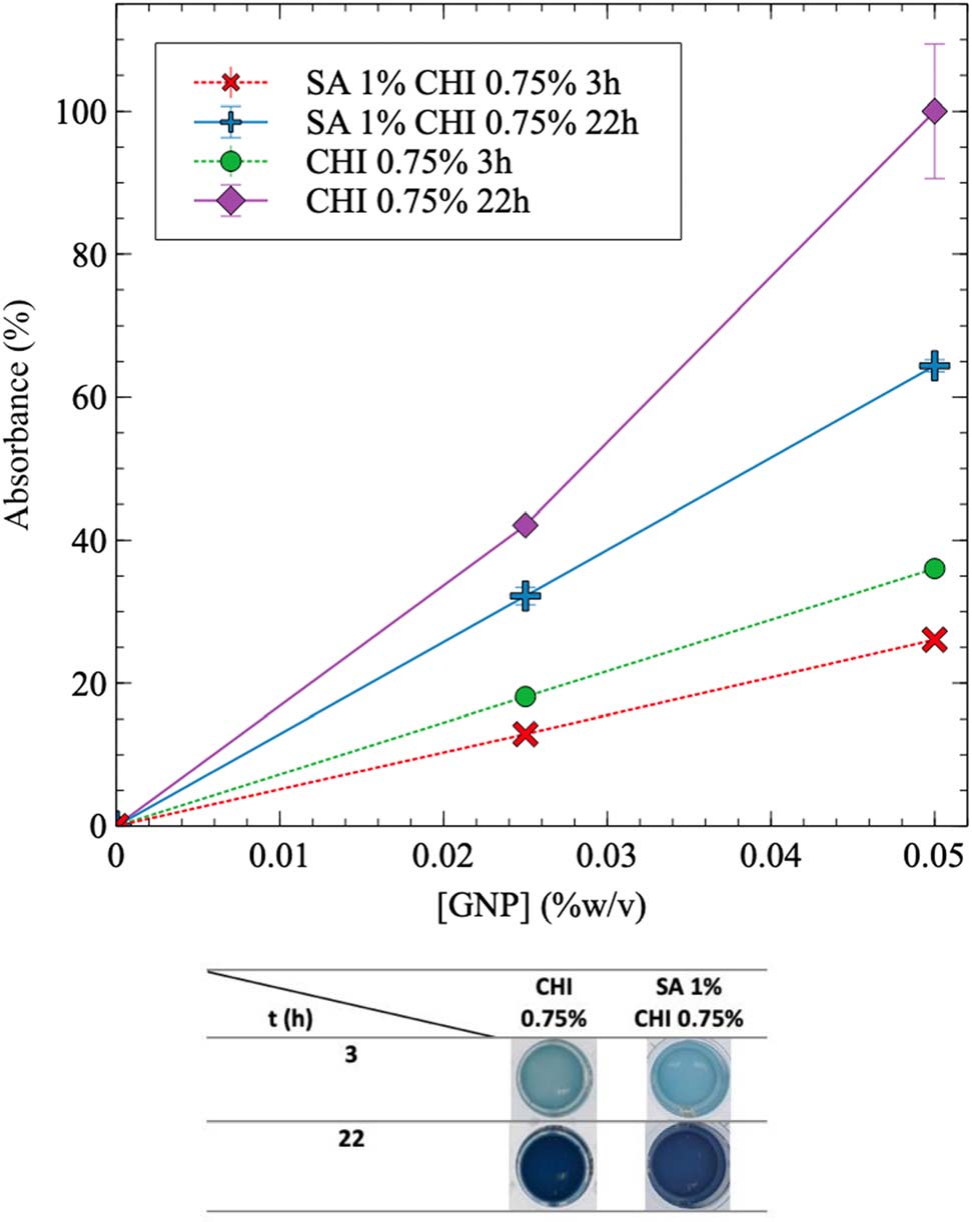
Kinetics of sodium alginate (SA)/chitosan (CHI) solutions crosslinked using genipin (GNP) and monitored by UV-vis spectroscopy. Absorbance as a function of GNP concentration and time, for both pure CHI (0.75% w/v), and SA 1% CHI 0.75% w/v starting solutions. For all experimental points, *N* = 3, and dotted lines are guides for the eyes. Some of the plate wells have been filled with water to prevent gel drying. After 22 h, gels were fully formed, and data at longer times are not shown.

The addition of SA into CHI gels was next investigated. Fig. 4 exhibits similar trends for SA/CHI gels, compared to pure CHI gels. However, the absorbance values at a given time are always lower for the SA/CHI formulations, compared to pure CHI (at similar CHI compositions). This is probably due to the slower GNP diffusion in solutions and gels also containing SA, slowing down the crosslinking reaction. To validate these observations, the gelation time was also quantified by rheometry on the same CHI and SA/CHI solutions.

#### 3.1.2 Rheometry

**Fig. 5:**
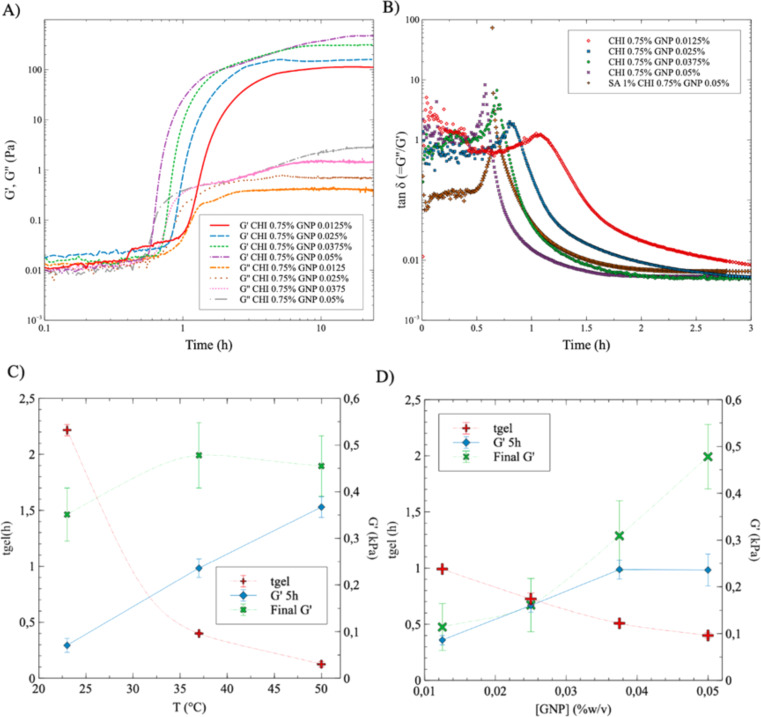
(A) Storage (*G*′) and loss (*G*′′) moduli for pure CHI gels (0.75% w/v) crosslinked with GNP, at a composition ranging from 0.0125% to 0.05% w/v, at 37 °C; (B) tan *δ* (=*G*′′/*G*′) as a function of time, from data in (A), compared to a solution composed of SA (1% w/v), CHI (0.75% w/v) and GNP (0.05% w/v); (C) gelation time (*t*_gel_), and storage modulus (*G*′) after 5 h of reaction time, and at equilibrium (at reaction completion), as a function of temperature *T*, for SA 1% CHI 0.75% GNP 0.05% w/v solutions; (D) gelation time (*t*_gel_), storage modulus (*G*′) after 5 h of reaction time, and at equilibrium at reaction completion, as a function of GNP concentration for SA 1% CHI 0.75% w/v at 37 °C.

Fig. 5A displays the value of the storage (*G*′) and loss (*G*′′) moduli as a function of time during the crosslinking reaction of pure CHI solutions (0.75% w/v), for GNP contents ranging from 0.0125% to 0.05% w/v. Whereas *G*′ and *G*′′ are comparable in magnitude initially – the solutions display viscoelastic behavior, *G*′ significantly increases compared to *G*′′ when the gelation point is reached, which is often associated to the intersection of the *G*′ and *G*′′ curves.^20^ At this point, *G*′ becomes significantly superior to *G*′′ and the material then behaves as a soft solid. Two trends are clearly observed: (1) the gelation point occurs earlier as the composition in GNP increases (the intersection point between *G*′ and *G*′′ shifts to the left, at an earlier time, as the GNP concentration increases), and (2) the *G*′ plateau value obtained at reaction completion, once it reaches a nearly stable value, increases as the GNP content increases (the plateau gradually increases as GNP concentration increases, since the density of crosslink points increases). Both trends are expected since more GNP means that the network can form more quickly, and gives a stronger gel at the end due to the higher crosslinks density.

The gelation point was next determined by plotting the values of tan *δ* (=*G*′′/*G*′) as a function of time and GNP composition (Fig. 5B). tan *δ* = 1 corresponds to the intersection of *G*′ and *G*′′ in Fig. 5A, and was used to determine the gelation time *t*_gel_ – *i.e.* when the storage modulus becomes equal to the loss modulus and the material starts to display a dominant soft solid-like behavior.^21^ As expected, the gelation time decreases with increasing GNP content, from 65 min ([GNP] = 0.0125% w/v) to 34 min ([GNP] = 0.05% w/v) (the maximum peak gradually shifts to the left, at earlier time, as the concentration in GNP increases). Interestingly, adding 1% w/v of SA (at a GNP content of 0.05% w/v) slighly increases gelation time – 39 min compared to 34 min without SA. This is also consistent with UV-vis results in Fig. 4, which shows that adding SA decreases the absorbance value at a given reaction time, supporting slower GNP diffusion and reaction with CHI.

The data displayed in Fig. 5C and D, extracted in part from Fig. 5A and B, exhibit similar trends as compared to UV-vis results: *t*_gel_ decreases with increasing *T*, from nearly 140 min down to 15 min as *T* increases from 22 °C to 50 °C (Fig. 5C for SA 1% CHI 0.75% GNP 0.05% w/v). *t*_gel_ also decreases with increasing GNP composition, from 105 min down to 35 min as the concentration of GNP increases from 0.0125% to 0.05% in SA 1% CHI 0.75% w/v gels at 37 °C. At a given reaction time, *G*′ is also higher at higher temperatures (Fig. 5C); however, when crosslinking is completed, the *G*′ values are comparable in magnitude for all three *T*. In addition, increasing the GNP composition leads to higher *G*′ values and shorter gelation times (Fig. 5D), consistent with the findings in Fig. 3. Similar trends and conclusions are observed when [CHI] = 0.5% (Fig. S4†).

**Fig. 6:**
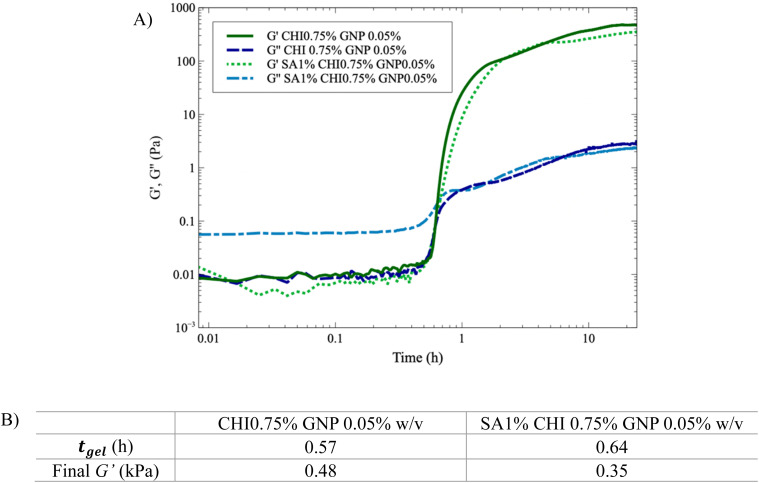
Impact of SA (1% w/v) addition on the crosslinking of CHI (0.75% w/v) with GNP (0.05%) at 37 °C. (A) *G*′ and *G*′′ as a function of time, with and without the addition of SA; (B) impact of SA addition on *t*_gel_ and final *G*′ value.

Fig. 6 shows that *t*_gel_ increases by nearly 15% with the addition of SA (1% w/v). In addition, *G*′′ is significantly higher initially for the solution containing SA (Fig. 6A), which slows down GNP diffusion and increases *t*_gel_. The presence of SA also leads to nearly comparable (or slightly lower) final *G*′ values. However, *G*′ still slowly increases when SA is added, due to the slower diffusion of genipin, in accordance with the absorbance results showing a lower value at a given time when SA is added (Fig. 4) – the absorbance should also continue to increase after 22 h. In comparison, when only CHI is present, both the absorbance and *G*′ have reached plateau values after 22 h (Fig. 3A, B and 6). Finally, when both the SA and the GNP concentrations are kept constant at 1% and 0.025% w/v respectively (Fig. S5A†), the final *G*′ and *t*_gel_ values do not show clear dependences on CHI composition and remain nearly constant. However, at 0.05% w/v of GNP (Fig. S5B†), the final *G*′ value significantly decreases as the composition in CHI increases from 0.5% to 0.75% w/v, whereas *t*_gel_ does not vary much. This is explained by the syneresis observed at higher GNP content, as Fig. 3B illustrates, combined with the higher amount of CHI.

### 3.2 Microstructure analysis of polymer molds and macroporous gels

**Fig. 7:**
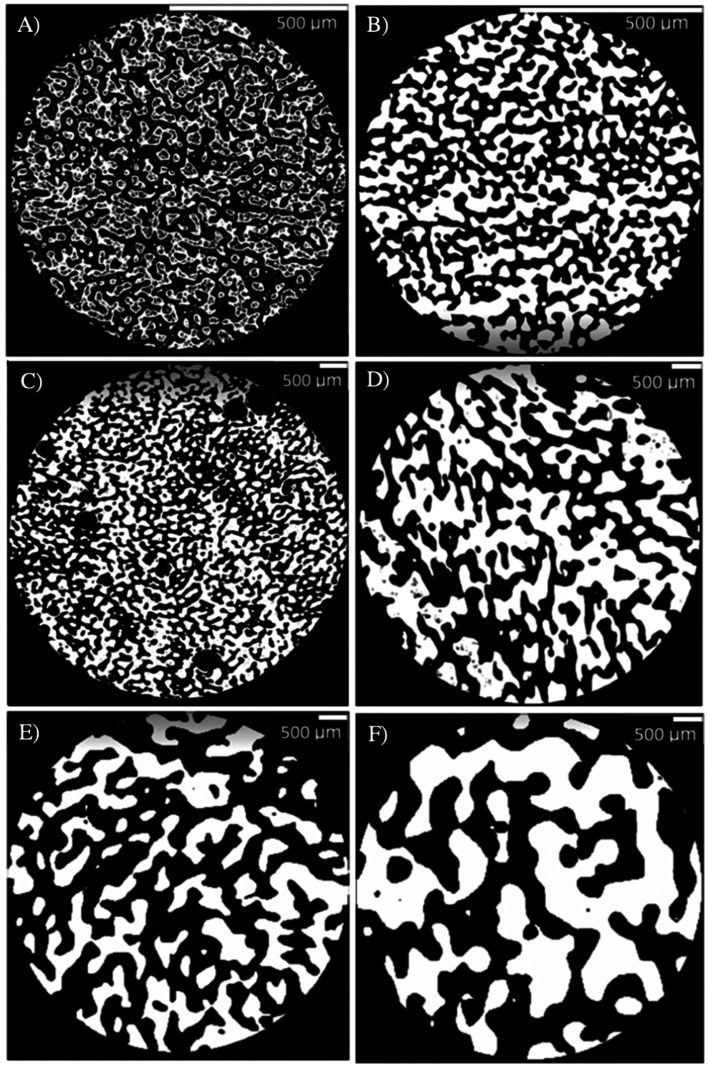
Cross-sections of porous PLA molds obtained by microCT for (A) 5, (B) 10, (C) 20, (D) 30, (E) 45 and (F) 60 min of quiescent annealing time (PLA: white domains; pores resulting from selective PS extraction: black domains). Note the difference between the scales of (A) and (B), compared to (C to F).

Quiescent annealing of the melt-processed co-continuous blend of PS and PLA (50/50 wt%) leads to the gradual coarsening of the microstructure, as the microCT micrographs in Fig. 7 illustrate. Coarsening of the microstructure occurs due to the interfacial tension between the PS and PLA phases (5.8 ± 0.6 mN m^−1^),^23^ as a way to reduce the specific interfacial area towards full phase separation. Porous PLA molds with a range of porosities are then obtained by selectively extracting the PS phase.

**Fig. 8:**
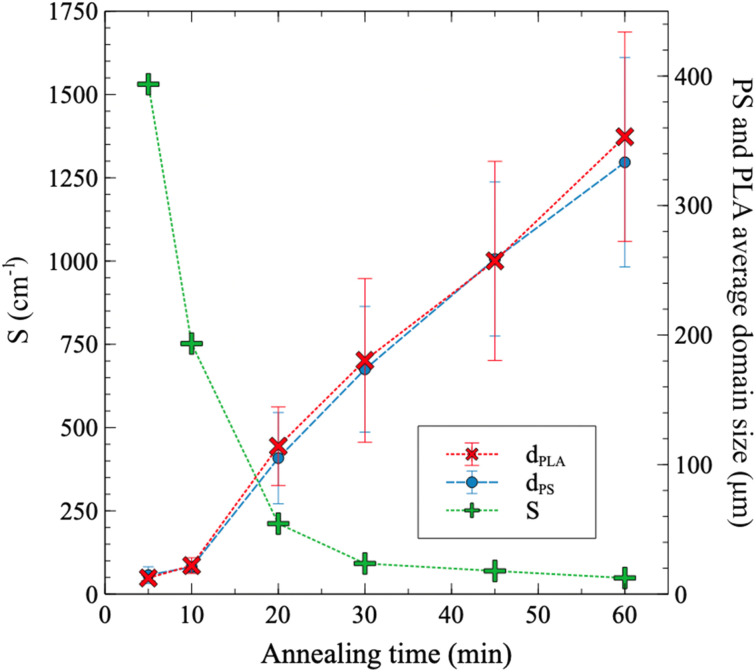
Extracted data from microCT analysis: PS and PLA domains sizes (*d*_PLA_ and *d*_PS_), and specific interfacial area (*S*) between the PS and PLA phases. The domain size at a given annealing time corresponds to the average of the size distribution, with the bars representing the width of the distribution (±1 standard deviation, modeled with a Gaussian distribution). The domain size distributions are provided in Fig. S6.†

For short annealing times, coarsening is slower due to thermal inertia of the samples (Fig. 8). However, over 10 min of annealing time, a quasi-linear trend is observed. MicroCT evaluation confirms that the volume fraction of PS and PLA domains closely resembles the composition of the original extruded blend, with both materials occupying approximately 50% of the total volume (Table S3†). The average sizes of the PS and PLA domains are nearly identical at a given annealing time – for PLA, it increases from 12 μm to 373 μm as annealing progresses from 5 min to 60 min. The specific interfacial area *S* decreases then from 1531 to 48 cm^−1^. This is consistent with values reported by Esquirol, Sarazin & Virgilio.^12^ Fig. S6† provides the pore size distributions as a function of annealing time, showing an evolution of the distribution towards higher values as annealing time increases.

**Fig. 9:**
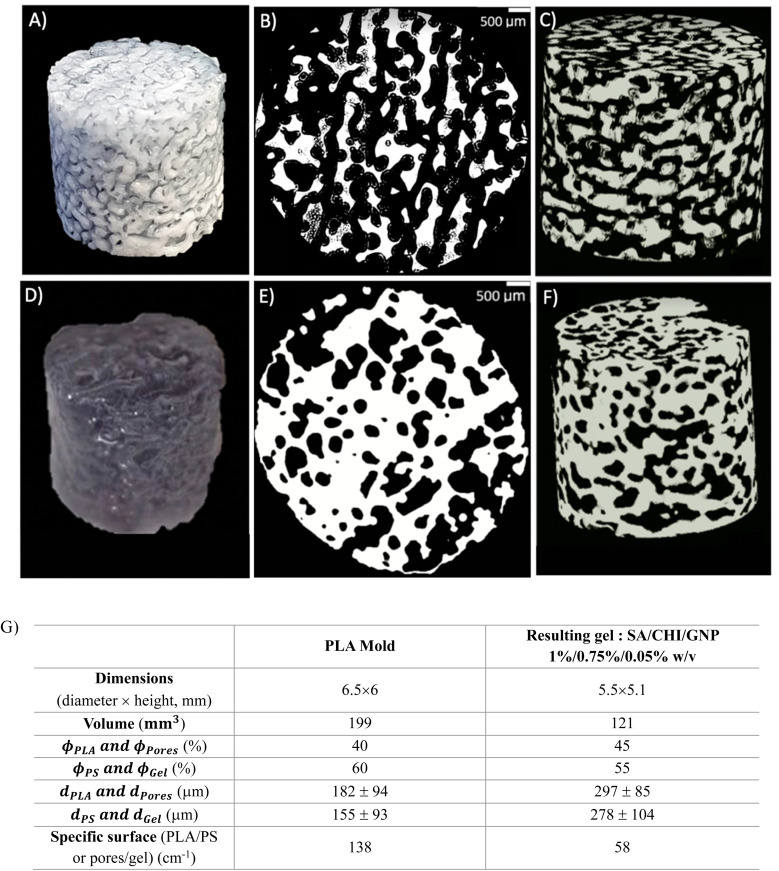
Injected mold (A to C) and resulting porous gel (D to F), (B, E) and (C, F) are 2D and 3D microCT reconstructions, and the extracted microstructural data are presented in (G).

Fig. 9 compares the microstructural features of a PLA mold (*t*_anneal_ = 30 min) to the resulting macroporous gel. During the molding process, the gel phase replaces the PS phase in the original polymer blend, whereas the pores in the gel come from the extraction of the PLA phase.

First, the porous gel retains the overall cylindrical shape of the mold, as Fig. 9A and D illustrate. However, the gel decreases in size compared to the mold (Fig. 9G): 15% of reduction for the dimensions, 40% reduction in volume, which is attributed to syneresis. The gel also exhibits larger pores compared to the original PLA domains (*d*_PLA_ = 182 μm *vs. d*_pores_ = 297 μm, recall that the PLA domains are extracted to yield the porous gel) – a similar feature is observed when comparing the average size of the gel domains, compared to the initial PS domains. This apparent increase in pore size could also be the result of gel syneresis, but also (and most probably) from the difficulty in completely removing the free water within the smaller pores before the microCT analysis, leading to an apparently larger average pore size. However, the porosity remains fully interconnected, as Fig. 9C and F show. For the sake of clarity in the following sections, the average size of the PLA domains will be used as the value of the average pore size in porous gels, since it was not possible to visualize the porosity within the gels with smaller pore sizes, as removing the water from the pores resulted in significant gel shrinking.

### 3.3 Mechanical properties in compression

There is a nearly linear relationship between the compression modulus of pure, bulk SA gels crosslinked with CaCl_2_, with SA concentration – from 16.8 kPa at 0.5% w/v of SA, to 95.4 kPa at 2% w/v SA (Fig. S7†). As expected, the modulus of SA porous gels (with an average pore size of 180 μm, prepared with PLA molds initially annealed during 30 min) decreases significantly compared to the bulk gels. At 0.5% w/v SA, the porous gels could not withstand the compression test, whereas the modulus at 1% and 2% w/v SA decreased by factors of approximately 4 and 4.2, at respectively 9.4 and 22.2 kPa (Fig. S7†).

For porous gels (average pore size of 180 μm), adding CHI to the formulation resulted only in a slight decrease of the compression modulus (not statistically significant, *p* = 0.1394), from 9.4 ± 1.4 kPa for SA 1% w/v, to 5.3 ± 1.6 kPa for SA 1% CHI 0.5% w/v (no GNP crosslinking) (Table S4†). Then, when CHI was also chemically crosslinked with GNP, the compression modulus increased from 5.3 to nearly 10 kPa, indicating that covalent crosslinking enhanced gel strength. Furthermore, two methods of crosslinking with GNP were evaluated: adding GNP to the SA/CHI solution before injection and gelation in the molds, or immersing the physically (Ca^2+^) crosslinked SA/CHI gels, following mold extraction, into an aqueous GNP solution – *i.e.* gelation *via* diffusion. Both methods resulted in similar compression moduli (Table S4†), demonstrating that either approach can be used, although the result was less significant for the diffusion-based process compared to GNP added before molding (*p* = 0.24 vs. p*).

The impact of CHI composition was also evaluated. A very slight increase for the porous gels modulus was observed, but no significant effect was noticed for bulk gels (Table S5†). A similar result was also obtained regarding the effect of GNP, since no significant difference was observed by increasing the GNP concentration from 0.025% to 0.1% (*p* > 0.05) (Table S6†). Overall, the concentration of SA, combined with CHI crosslinking, are the two factors having the most significant effects on the compression modulus, whereas the effects of CHI and GNP concentrations do not significantly impact the resulting modulus, at least over the investigated ranges of concentrations.

**Fig. 10:**
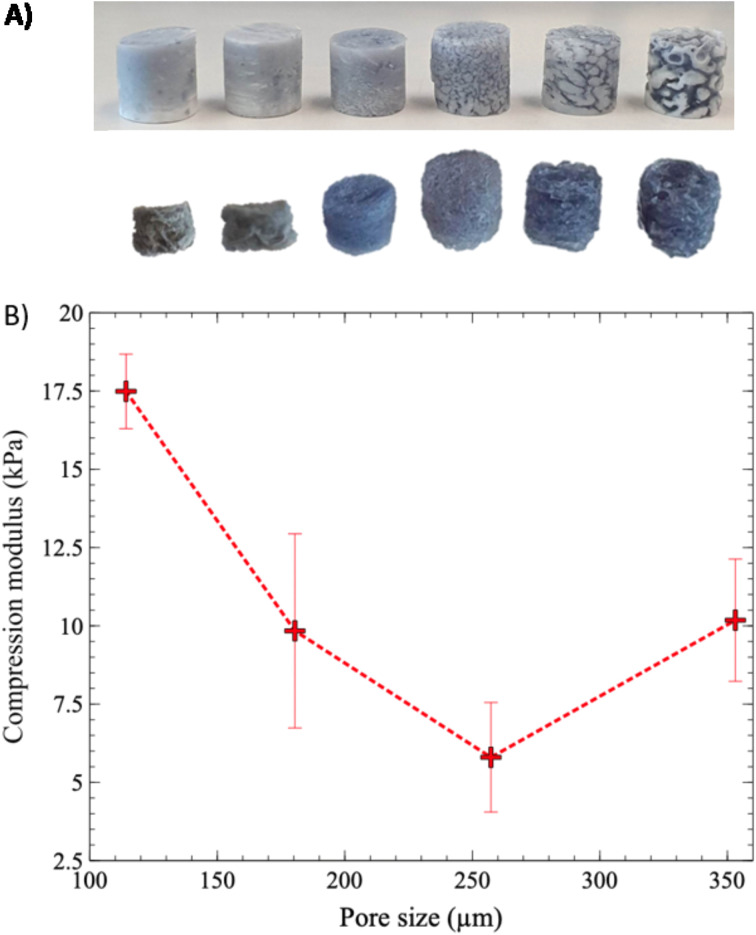
Appearance (A) and compression modulus (B) of porous hydrogels as a function of average pore size. (A) PLA molds filled with hydrogels (top) and resulting porous SA/CHI/GNP gels (bottom) after PLA extraction; (B) compression modulus as a function of pore size, for SA 1% CHI 0.75% GNP 0.05% w/v gels.

Next, the relationship between the value of the compression modulus and average pore size was investigated, over the whole range of average pore sizes, from 12 to 373 μm (Fig. 10). Gels with the smallest pore sizes, 12 and 22 μm, corresponding to annealing times of 5 and 10 min, exhibited significant size reduction and deformation after the extraction of the PLA phase (Fig. 10A), and it was impossible to accurately measure their compression moduli. Although the exact causes are still not clear, excessive syneresis, or crosslinking under strong confinement conditions, could be involved and remains to be fully investigated.

Starting from 20 min of annealing time, the compression modulus of the gels showed a decreasing trend, from 17.5 kPa at an average pore size of 114 μm, to 5.8 kPa at 257 μm (Fig. 10B). However, an increase in the compression modulus was observed when the pore size increased to 353 μm. In a recent work, Zhang *et al.* (2020) demonstrated a similar behavior for polymer foams.^24^ At small pore size values, the compression modulus first decreases rapidly as pore size increases. Then, this sharp decrease levels off and is followed by a slower one as pore size continues to increase. Their numerical modeling experiments demonstrated that at small pore sizes, the stress concentration is more equally distributed, and the porous structure becomes “self-reinforcing”. At larger pore sizes, however, stress concentrates in the walls or struts, until failure occurs.

Interestingly, nearly all tested formulations yielded porous gels with a compression modulus within the targeted range of 1–10 kPa, corresponding to the properties of soft brain tissues. This is useful since it could provide some flexibility regarding the formulation when assessing other properties such as colonization by GBM cells and their accumulation as a function of pore size, without impacting significantly the mechanical properties.

### 3.4 Porous gel stability in aqueous medium

The dimensional and mechanical stability of the gels in four different aqueous media at 37 °C were assessed next over 15 days. The results in CaCl_2_ solutions, without and with antibiotics, are not shown as no significant change in gel integrity, dimensions, or compression modulus were observed, and thus served as references.

**Fig. 11:**
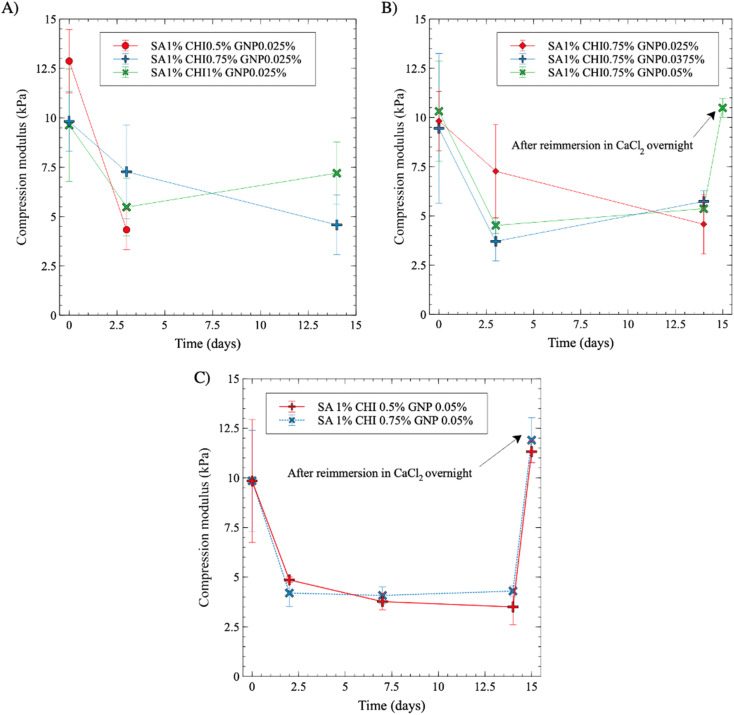
Evolution of compression modulus of porous gels (average pore size of 180 μm) in PBS medium: (A) effect CHI composition at constant SA and GNP contents (1% and 0.025% w/v, respectively); (B) effect of GNP composition at constant SA and CHI contents (1% and 0.75% w/v, respectively). (C) Evolution of compression modulus in DMEM medium, for 1% w/v SA, (0.5% or 0.75% w/v) CHI, 0.025% w/v GNP. Tests were realized at 37 °C, and solutions were changed after each measurement.

In PBS media supplemented with antibiotics, the compression modulus initially undergoes a noticeable decrease by more than 50% after 3 days, and then remains relatively stable over time (Fig. 11A). At constant SA and GNP contents (1% and 0.025% w/v, respectively), increasing the CHI content from 0.5% to 1% w/v slightly lowers the initial compression modulus, from nearly 12.5 kPa to 10 kPa, as reported in Fig. 11A. The modulus however decreased less sharply as the content in CHI increased. After 3 days, the gels with the lowest content in CHI, at 0.5% w/v, became too soft for further testing (see also Fig. S8A† for visual aspect of the gels). At higher contents however, the gels remained stable and could be handled for mechanical testing, and displayed stable modulus afterwards, demonstrating the stabilization impact of crosslinked CHI.

Increasing the GNP composition further did not lead to a significant improvement in gel stability after 15 days in PBS, as the compression modulus values are close and comprised between 4.6 and 5.7 kPa for GNP contents ranging from 0.025% to 0.05% w/v (p* for GNP 0.0375% and *p* > 0.05 for GNP 0.05% w/v between the first and 14th day) (Fig. 11B). However, it is worth noting that after 3 days in PBS, the composition with the lowest GNP content (0.025%) exhibited a comparatively smaller decrease of its compression modulus. Then, upon immersion in a CaCl_2_ solution on the 15th day, the gels regained their initial compression modulus of 10.4 kPa.

A similar decreasing trend is observed when the gels are immersed in DMEM medium (Fig. 11C and S8B† for visual aspect). SA 1% CHI 0.75% GNP 0.05% w/v gels appear to stabilize slightly more rapidly compared to a 0.5% w/v content in CHI. In addition, all gels maintained their integrity in DMEM – even at 0.5% CHI, which was not the case in PBS. Again, plunging back the gels after 15 days into a CaCl_2_ solution resulted in a full recovery of gel strength, at respectively 11.6 and 11.9 kPa for 0.5% and 0.75% w/v CHI contents.

The dimensions of the gels also changed in PBS and DMEM medium (Fig. S9†). First, each gel decreases in size following the extraction of the mold, as illustrated previously in Fig. 9. Specifically, gel thickness decreases from 6 mm to 5.1 mm, and the diameter decreases from 6.5 mm to 5.2 mm. When the gels are placed in PBS (Fig. S9A†), the dimensions further decrease, and stabilize after 3 days at a diameter of 4.8 mm and a thickness of 4.5 mm (p* and p**, respectively, between day 1 and day 3). On the other hand, when the gels are placed in DMEM medium (Fig. S9B†), the dimensions only slightly decrease, and they stabilize at a diameter of 5.2 mm and a thickness of 5.1 mm (*p* > 0.05). As a result, the effects of DMEM are milder on gel integrity and mechanical properties, compared to PBS, and CHI crosslinking clearly helps to stabilize the materials.^21^

### 3.5 Impact of irradiation on porous gels compression modulus

The effect of gamma irradiation dose on the mechanical properties was also assessed. The results are presented in Table 1. The dose was gradually increased from 10 to 30 Gy, which is considered as the superior limit for the treatment of GBM cancer. Overall, irradiation did not significantly alter or degrade the macroscopic aspects and mechanical properties of the gels (*p* > 0.05 for all doses compared to non-irradiated gels). In addition, no delayed impact was observed as the modulus remained unchanged one week after irradiation. When placed on absorbent paper, water could easily be absorbed, suggesting that the pores were still open and accessible.

**Table 1:** Compression modulus of porous gels after exposure to increasing doses of gamma irradiation (SA 1% CHI 0.75% GNP 0.025% w/v, average pore size = 180 μm) (*N* = 4)

Dose (Gy)	0	10	15	20	30	30 (1 week after irradiation)
Compression modulus (kPa)	9.9 ± 2.5	10.2 ± 2.4	8.3 ± 1.0	9.9 ± 1.3	9.0 ± 2.4	10.0 ± 6.0

### 3.6 Impact of hydrogel formulation on the fluorescent properties of mCherry expressed by the F98 cells

The products of GNP reaction with primary amine groups are known to fluoresce in the 380–700 nm wavelength region. When GNP crosslinks CHI, the maximum excitation and emission wavelengths are measured at 550 nm and 650 nm, respectively.^25^ An overlap with the fluorescence properties of mCherry expressed by F98 cells is expected since its maximum excitation wavelength *λ*_ex_ = 530 nm, while *λ*_em_ = 590 nm. The extent of interference with the fluorescence expressed by the F98 mCherry cells has been determined in the presence of three different types of hydrogel formulations: SA 1% CHI 0.75% GNP 0.05% w/v, and those formulated with SA 1% CHI 0.75% (no GNP), and SA 1% only (Fig. S10†). All three were grafted with the RGD adhesion peptide and have similar average pore sizes (180 μm). Our results show that in SA 1% hydrogels, the fluorescence of the F98 mCherry cells is proportional to the number of cells (Fig. S10A†). On the other hand, slight fluorescence was measured due to the addition of CHI even without cells (Fig. S10B†), whereas a very high fluorescence intensity was observed in the absence of F98 mCherry cells in hydrogels containing CHI crosslinked by GNP (almost 8 times higher than the value observed when adding 1 × 10^6^ F98 mCherry cells to 1% SA hydrogels) (Fig. S10C†). This intensity gradually decreased while adding F98 mCherry cells to reach a stable value of around 60 000. These results confirm the interference of the fluorescing product of the crosslinking reaction between CHI and GNP, and the fluorescence of the mCherry marker.

### 3.7 Accumulation and retention of F98 cells in hydrogels

**Fig. 12:**
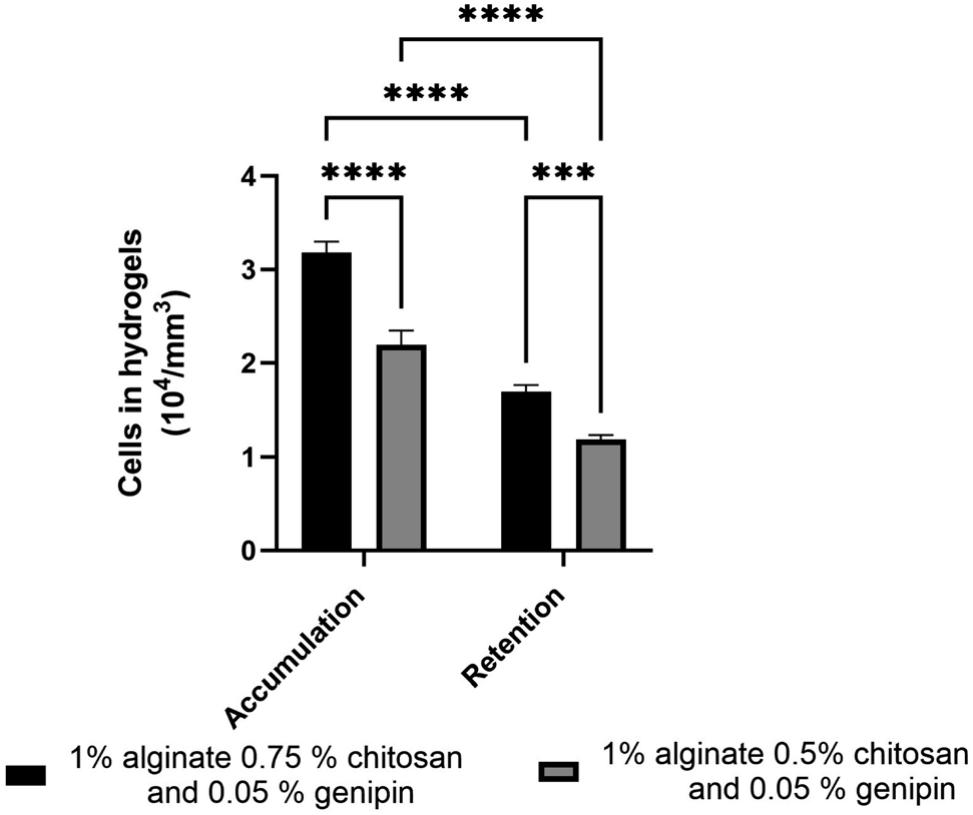
Accumulation and retention of F98 mCherry cells in hydrogels formulated with SA 1% CHI 0.75% GNP 0.05% w/v (in black) and SA 1% CHI 0.5% GNP 0.05% w/v (in grey).

The accumulation and retention of F98 cells in the hydrogels were then quantified using the Alamar Blue reagent, which measures the metabolic activity of cells present in the hydrogels. After 24 h of incubation, the number of F98 cells accumulated in the hydrogels was determined after gentle washing with PBS buffer first. The number of retained cells was then determined afterwards following a stronger wash.^11^ For this test, two different types of hydrogels were used: SA 1% CHI 0.5% GNP 0.05% w/v, and SA 1% CHI 0.75% GNP 0.05% w/v, to determine which formulation would optimize F98 cellular accumulation and retention (Fig. 12).

Hydrogels composed of SA 1% CHI 0.75% GNP 0.05% w/v significantly accumulated (p****) and retained (p***) more F98 cells. In fact, the accumulation as well as the retention of F98 cells were 1.4 times higher in SA 1% CHI 0.75% GNP 0.05% hydrogels, compared to SA 1% CHI 0.5% GNP 0.05% gels. These increases could be associated with a higher positive charge when the concentration of CHI increases from 0.5% to 0.75%, which favors the adhesion of negatively charged F98 cells. However, cellular retention decreased by 1.8% in both hydrogels, suggesting that not all F98 cells were firmly adhered to the gels after 24 h of incubation.

### 3.8 Distribution of F98 cells within the hydrogels

The distribution of F98 cells in the hydrogels was measured at four different levels (L1 to L4) ranging from 1 mm to 3.5 mm deep, starting from the top of the hydrogels. Two hydrogel formulations were tested: SA 1% CHI 0.5% GNP 0.05 w/v, and SA 1% CHI 0.75% GNP 0.05% w/v. Images at the different levels, for both formulations, are shown in Table 2. F98 cells accumulated in the hydrogels were fixed and labeled, and their distribution was assessed using the epifluorescence microscope EVOS FL Auto. The images obtained were then analyzed using a home-made algorithm which allows to determine the number of cell clusters and their average size at each level, based on their fluorescence intensity at the focal point.^11^ The fluorescence emitted by the cell clusters diffuses through the hydrogel which leads to their detection at different levels L, as illustrated by the red rectangles drawn on levels L1 to L3 (Table 2). In order to count a cell cluster only once, only those detected at the focal point of a level are taken into account. The number of cells in a cluster is determined approximately based on the diameter of each cluster and the average diameter of an individual cell. For example, a cell cluster of 200 μm in diameter contains approximately 1000 cells with a diameter of 20 μm.

**Table 2:** Representative distribution of F98 cells at 4 different levels (L1–L4) in SA 1% CHI 0.75% GNP 0.05% w/v, and SA 1% CHI 0.5% GNP 0.05% w/v hydrogels. Red squares show in focus and out of focus cell clusters as a function of the considered level^a^

1% alginate 0.75% chitosan 0.05% genipin	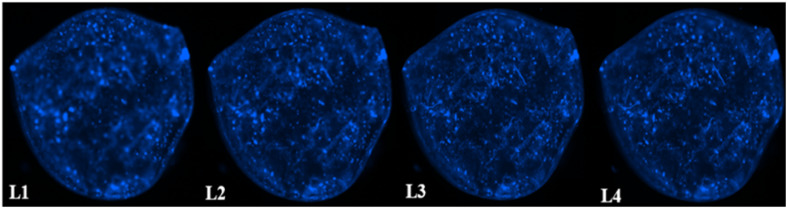
1% alginate 0.5% chitosan 0.05% genipin	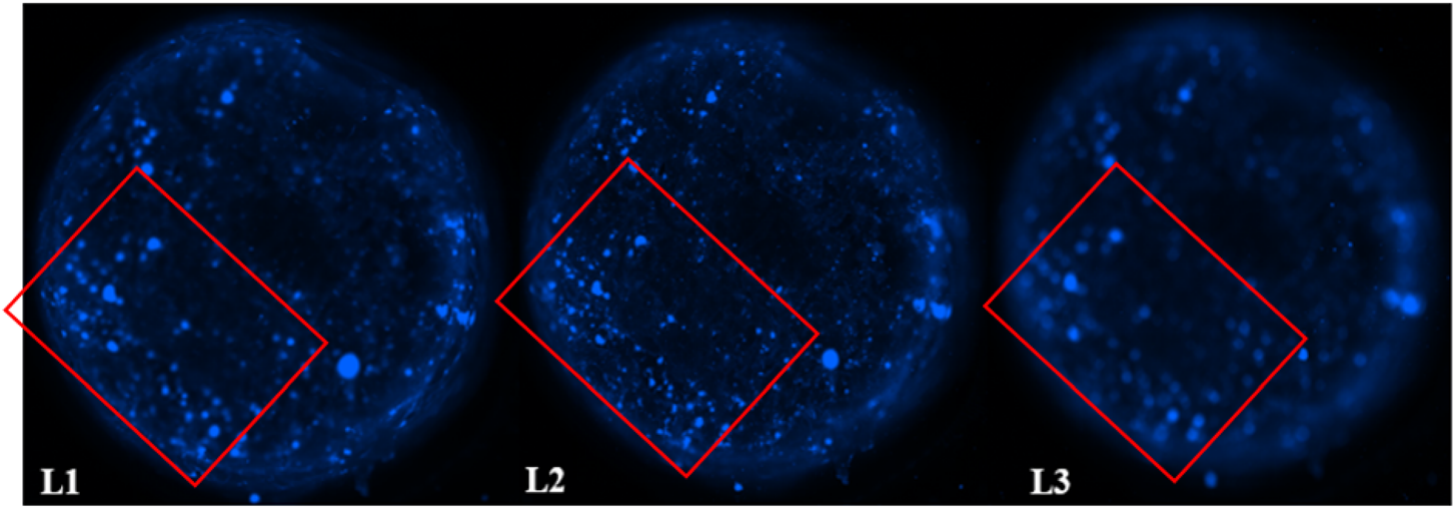

a At a given level, cells at the focal point (located in the observation plane) are sharply defined and bright blue, whereas cells that are out of focus (*e.g.* deeper in the gel) also display less intensity, explaining the difference in fluorescence.

**Fig. 13:**
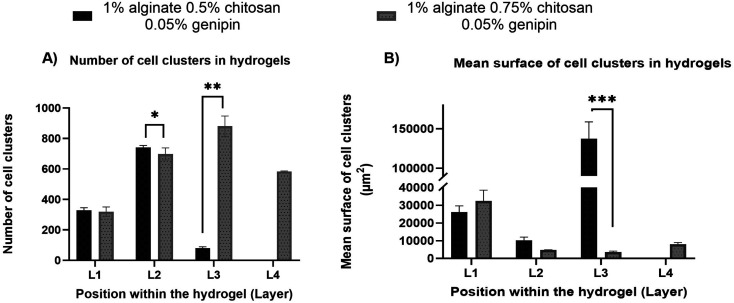
(A) Number of cell clusters within levels L1 to L4 in hydrogels formulated with SA 1% CHI 0.5% GNP 0.05% w/v, and in SA 1% CHI 0.75% GNP 0.05% w/v; (B) mean surface of cell clusters in levels L1 to L4, in hydrogels formulated with SA 1% CHI 0.5% GNP 0.05% w/v, and in SA 1% CHI 0.75% GNP 0.05% w/v.

As mentioned above, in the SA 1% CHI 0.5% GNP 0.05% hydrogels, the cells stopped migrating once they reached level L3, while they reached level L4 in the SA 1% CHI 0.75% GNP 0.05% hydrogels. The results also show that by increasing the concentration of CHI from 0.5% to 0.75%, twice as more cell clusters are observed, supporting the role of the positively charged CHI in promoting adhesion of F98 cells (Fig. 13A), a result that was also observed with cell accumulation and retention assays (Fig. 12). In addition, the mean surface area of the cell clusters at the 4 levels in hydrogels containing 0.75% CHI were 3 times higher compared to clusters in gels containing 0.5% CHI (Fig. 13B).

Interestingly, in SA 1% CHI 0.5% GNP 0.05% hydrogels, the highest cell clusters average surface area was observed at level L3. This observation is consistent with the cessation of cell migration at this level, which could be associated with the larger size of the cell clusters. Conversely, in hydrogels containing 0.75% CHI, the average size of cell clusters at this level was 38 times smaller, correlating with their migration down to level L4 (Fig. 13B). To conclude, these results show that hydrogels composed of SA 1% CHI 0.75% GNP 0.05% yields a more homogeneous distribution of F98 cells.

### 3.9 Discussion

Chitosan is currently used or considered for a variety of biomedical applications, including drug delivery, wound dressing, regenerative medicine, *etc.*^26–28^ This work improves on the previous results of Safi *et al.* (2022),^11^ which demonstrated that the addition of CHI in SA based gels indeed delayed, but did not stop, gel dissolution in media containing electrolytes (*i.e.* Na+ ions) such as cell culture media, and especially PBS. In this case, CHI interacted electrostatically with SA, but was not chemically crosslinked, and the gels displayed very fine but uncontrolled precipitates of SA and CHI. The main objective of the present work was then to develop homogeneous, chemically crosslinked and macroporous SA/CHI gels to enhance their stability in cell culture medium. This was achieved by combining (1) the addition of sodium bicarbonate to the initial CHI solution, followed by the addition of the SA solution,^16^ and (2) by subsequently crosslinking CHI with GNP. The addition of sodium bicarbonate allowed the mixing of both CHI and SA in solution at alkaline pH, without the formation of uncontrolled precipitates. By decreasing the pH again, it was possible to control their interaction in solution, yielding clear hydrogels. Although we only tested one particular grade of CHI (DDA of 85%, similar to Komoto *et al.*'s work^16^), we believe this approach could be extended to a wider selection of CHI grades.

Interestingly, the gelling kinetics can be monitored both by rheology, but also by UV-vis spectroscopy, as Fig. 3 to 6 demonstrate, due to the bluish color associated to the GNP crosslinking reaction. Controlling the gelling kinetics then allows the preparation of porous gels by molding in porous PLA templates. The resulting compression modulus of the porous gels are comparable to the values obtained previously by Safi *et al.* (2022), at ≈10 kPa, with significantly improved stability.^11^

Next, the combination of alginate and chitosan also offers a number of advantages. Alginate and chitosan bear different functional groups, *i.e.* hydroxyl and carboxylic acid groups for alginate, primary amine groups for chitosan. This variety of functional groups allow for multiple functionalization strategies, and complementary gelling mechanisms. For example, alginate forms hydrogels *via* complexation between its carboxylic acid groups and Ca^2+^ ions in solution. In addition, we functionalize alginate with RGD adhesion peptides to promote cell adhesion, *via* EDC/NHS chemistry (other specific peptides could then be grafted following this approach). On the other hand, chitosan can also be crosslinked covalently with genipin, a natural crosslinker, increasing the stability of hydrogels in physiological solutions, as Fig. 11 demonstrates. In addition, chitosan also promotes the adhesion of F98 glioblastoma cells,^11^ and could be functionalized *via* its primary amine groups (although we do not exploit this feature, this is an additional possibility).

When combined together, alginate and chitosan, bearing complementary positive and negative charges, can also improve gel stability *via* strong electrostatic interactions.^11,20^ Furthermore, genipin not only crosslinks chitosan, it also displays anti-inflammatory, anticancer, antithrombotic and antibacterial properties.^17^ Overall, the combination of alginate and chitosan offers a high level of flexibility both in terms of gelling mechanism and functionalization, *via* relatively simple and biocompatible chemistries, illustrating the advantages of alginate–chitosan hybrid materials.

The gel chemistry developed in this work respects the cancer cell trap requirements, with all selected components (biopolymers and crosslinking agent) being natural to ensure biocompatibility of the gels. The optimal formulation comprises 1% SA, 0.75% CHI, and 0.05% GNP w/v, for which no syneresis was observed for bulk gels. However, a 40% reduction in volume was observed when preparing macroporous gels (Fig. 9). We identify three possible causes: (1) the interface between the gel and the aqueous solution is quite higher for macroporous gels compared to bulk gels, which could exacerbate syneresis. Fig. 10 supports this, since volume reduction increases as the average pore size decreases – all gels were stored in water at equilibrium, showing that the water holding capacity decreases with decreasing average pore size; (2) the extraction of the polymer molds in chloroform could also solubilize or “extract” a fraction of water, leading to an irreversible volume reduction; (3) as the average pore size decreases, gelling is increasingly confined – whether this could have an effect remains to be investigated. Finally, note that all three phenomena could also work in tandem.

Gels displaying an average pore size of 180 microns, chosen to ensure an effective cellular infiltration, exhibit a compression modulus similar to brain tissues (1–10 kPa) both immediately after preparation, and after immersion in PBS and cell culture medium (rich in ionic species) for 15 days. They are also resistant to radiation and capable of accumulating and retaining F98 mCherry cells throughout their entire volume (Fig. 12 and 13). Indeed, the accumulation and retention properties are also quite similar to the previous results reported by Safi *et al.*,^11^ and support previous results about the importance of CHI in promoting a more homogeneous distribution throughout the gels (Table 2 and Fig. 13). However, the bluish color induced by GNP creates interference with the fluorescence measurements of F98 mCherry cells. In addition, avoiding the use organic solvents (herein chloroform) to dissolve the polymer molds (herein PLA) would eliminate the possibility of any residual solvent in the macroporous hydrogels (although we did not detect any). Future works will focus on substituting GNP chemical crosslinking, by a reaction avoiding the development of such coloration (while maintaining biocompatibility), and on the development of water-soluble polymer molds to avoid the use of organic solvents, and the undesired syneresis observed for macroporous hydrogels. This will facilitate the next step to evaluate the capacity of these gels to attract, using chemoattractants such as CXCL12,^29^ and accumulate GBM cells – and other variety of cells – in an *in vivo* rat model.

## 4. Conclusion

In this work, we have developed a sodium alginate/chitosan gel formulation to prepare homogeneous macroporous hydrogels, chemically crosslinked with genipin, and comprising fully interconnected pores with an average size of 180 microns. The best results were obtained with a formulation of SA 1% CHI 0.75% GNP 0.05% w/v. Indeed, chemical crosslinking of CHI by GNP yields a compression modulus of 10.3 kPa. This resulted in enhanced gel stability in cell culture medium rich in ionic species, with a compression modulus of 4.3 kPa after 14 days in DMEM medium. In addition, a radiation dose of 30 Gy had no significant impact on the mechanical properties and stability of the gels, even one week after irradiation. F98 mCherry cells distributed, accumulated and were retained in the whole gel volume.

## Data availability

The data supporting this article have been included as part of the ESI† file, which contains: genipin (GNP) compositions and chitosan (CHI)/GNP ratios in gels; microstructural parameters of polystyrene/polylactide blends; mechanical compression properties of bulk and porous gels; molecular structures of chitin, chitosan and sodium alginate; absorbance master curve of CHI crosslinked with GNP; absorbance (UV-vis) of CHI/GNP gels; gelation time and storage modulus (*G*′) of sodium alginate (SA)/CHI/GNP gels measured by rheometry; pore size distributions of porous polylactide molds as a function of annealing time; stability of gels in PBS and DMEM medium; F98 mCherry cells fluorescence intensity in hydrogels (PDF).

## Author contributions

Lauriane Parès: conceptualization, methodology, validation, formal analysis, investigation, writing – original draft, visualization. Sahar Naasri: methodology, validation, formal analysis, investigation, writing – original draft, visualization. Lisa Delattre: methodology, validation, formal analysis. Hélène Therriault: methodology, validation, formal analysis. Benoît Liberelle: methodology, validation, formal analysis. Gregory De Crescenzo: resources, writing – review & editing, funding acquisition. Marc-Antoine Lauzon: conceptualization, writing – review & editing, funding acquisition. Nathalie Faucheux: conceptualization, writing – review & editing, funding acquisition. Benoit Paquette: conceptualization, methodology, formal analysis, resources, writing – review & editing, supervision, project administration, funding acquisition. Nick Virgilio: conceptualization, methodology, formal analysis, resources, writing – review & editing, supervision, project administration, funding acquisition.

## Conflicts of interest

There are no conflicts to declare.

## Supplementary Material

RA-014-D4RA06197G-s001
