# Supplementary material for: Macroporous chitosan/alginate hydrogels crosslinked with genipin accumulate and retain glioblastoma cancer cells
Source: RSC Adv. 2024 Nov 5;14(48):35286–304. doi: 10.1039/d4ra06197g (PMC11537210; doi:10.1039/d4ra06197g)
Supplement: RA-014-D4RA06197G-s001 [file RA-014-D4RA06197G-s001.pdf]

Supplementary Information (SI) for RSC Advances.
This journal is © The Royal Society of Chemistry 2024

Supporting Information

Macroporous Chitosan/Alginate Hydrogels Crosslinked with Genipin Accumulate and Retain Glioblastoma Cancer Cells

Lauriane Parès,[a] Sahar Naasri,[b] Lisa Delattre,[a] Hélène Therriault,[b] Benoît Liberelle,[c] Gregory De Crescenzo,[c] Marc-Antoine Lauzon,[d] Nathalie Faucheux,[d] Benoit Paquette[b] and Nick Virgilio[a]

[a] Research Center for High Performance Polymer and Composite Systems (CREPEC), Department of Chemical Engineering, Polytechnique Montréal, Montréal, Québec, H3C 3A7, Canada.

[b] Center for Research in Radiotherapy, Department of Nuclear Medicine and Radiobiology, Faculty of Medicine and Health Sciences, Université de Sherbrooke, Sherbrooke, Québec, J1H 5N4, Canada.

[c] Department of Chemical Engineering, Polytechnique Montréal, Montréal H3C 3A7, Québec, Canada.

[d] Department of Chemical and Biotechnological Engineering, Faculty of Engineering, Université de Sherbrooke, Sherbrooke, Québec, J1K 2R1, Canada.

Table S1. GNP compositions and CHI/GNP ratios in solutions and gels containing 0.75% w/v CHI.

GNP (%w/v)	0.0125	0.025	0.0375	0.05
CHI / GNP ratio	60	30	20	15

Table S2. GNP compositions and CHI/GNP ratios in solutions and gels containing 0.5% w/v CHI.

GNP (%w/v)	0.025	0.0375	0.05	0.075
CHI / GNP ratio	20	13.33	10	6.67

Table S3. Extracted data from microCT analysis for the PS and PLA domains: volume fractions ϕ, average domain sizes d and specific interfacial area S.

Annealing time (min)	5	10	20	30	45	60
ϕ_{PLA}	41	41	49	48	45	49
ϕ_{PS}	59	59	501	52	55	52
d_{PLA}	12 $\pm$ 2	22 $\pm$ 6	114 $\pm$ 30	187 $\pm$ 63	256 $\pm$ 77	373 $\pm$ 81
d_{PS}	15 $\pm$ 6	21 $\pm$ 4	105 $\pm$ 35	174 $\pm$ 49	259 $\pm$ 60	333 $\pm$ 81
Specific surface PLA (cm^{-1})	1531	752	211	92	70	48

Table S4. Porous gel mechanical properties in compression: effect of genipin crosslinking before VS after crosslinking with $CaCl_2$, ($N = 4$)

	SA 1%	SA 1% CHI 0.5%	SA 1% CHI 0.5% GNP 0.05%	SA 1% CHI 0.5% GNP 0.05% by diffusion
Compression modulus (kPa)	9.4 $\pm$ 1.4	5.3 $\pm$ 1.6	9.8 $\pm$ 3.1	8.2 $\pm$ 2.2

Table S5. Bulk and porous hydrogels (180 μm average pore size) compression modulus (kPa) as a function of SA and CHI contents, at 0.025% w/v GNP content.

	SA 0.5% CHI 1%	SA 1% CHI 0.25%	SA 1% CHI 0.5%	SA 1% CHI 0.75%	SA 1% CHI 1%
Bulk gels	11.5 $\pm$ 1.0	26.3 $\pm$ 9.9	20.5 $\pm$ 8.5	24.3 $\pm$ 9.4	Not tested
Porous gels	Can't stand	9.9 $\pm$ 1.6	12.6 $\pm$ 2.4	8.7 $\pm$ 2.4	9.8 $\pm$ 2.2

Table S6. Compression modulus (kPa) of porous gels (180 μm average pore size) as a function of GNP and CHI compositions.

[GNP] (% w/v)	SA 1% CHI 0.5%	SA 1% CHI 0.75%	SA 1% CHI 1%
0.025	12.5 $\pm$ 2.4	8.7 $\pm$ 2.4	9.8 $\pm$ 2.2
0.0375	6.6 $\pm$ 2.5	9.5 $\pm$ 3.8	7.3 $\pm$ 1.8
0.05	9.8 $\pm$ 3.1	10.3 $\pm$ 2.6	Not tested
0.1	7.6 $\pm$ 2.0	Not tested	Not tested

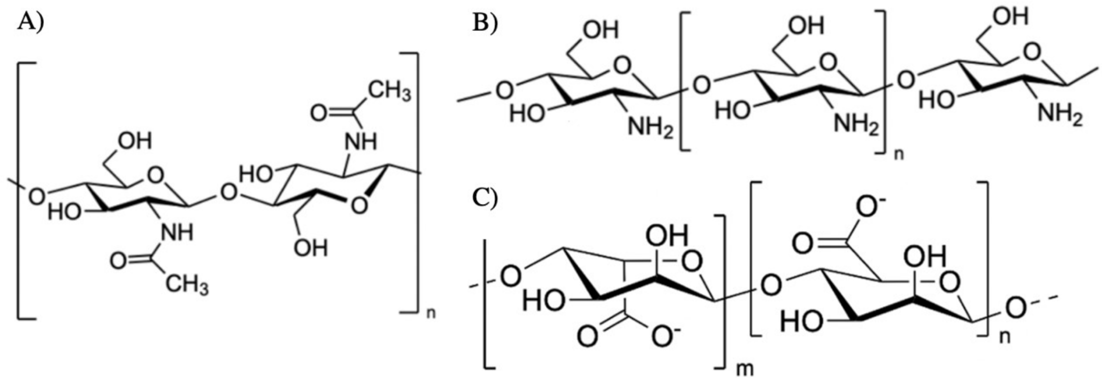

Figure S1. Molecular structure of A) chitin B) chitosan and C) sodium alginate.

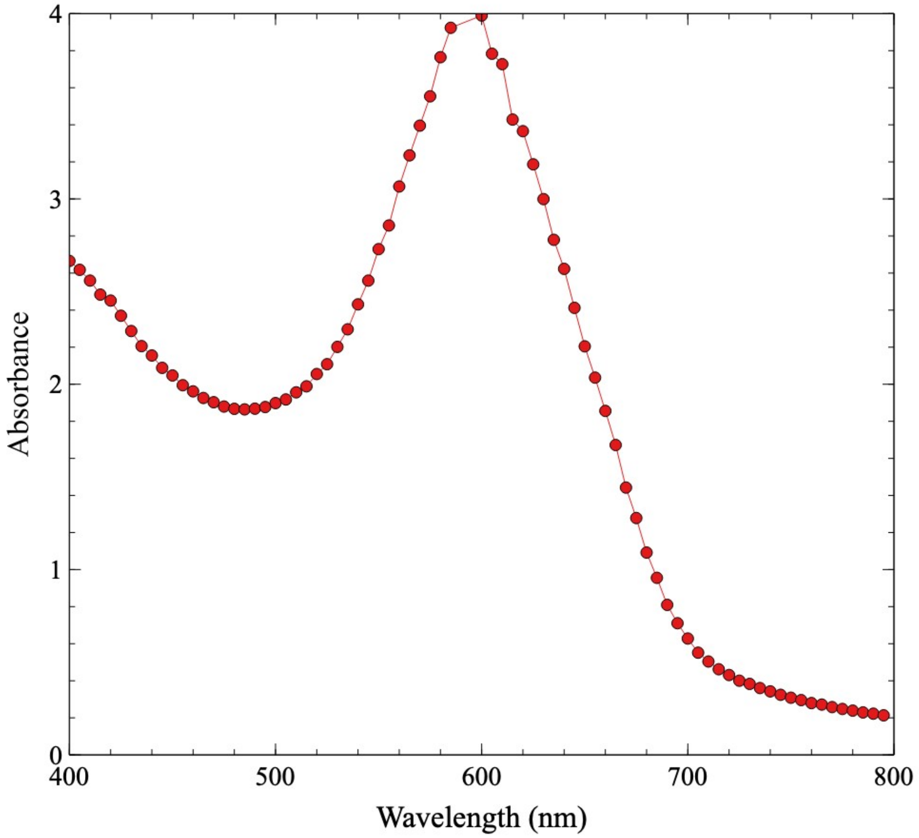

Figure S2. Absorbance master curve of CHI 0.75% GNP 0.05% w/v after 24h of gelation time at 37 °C in a plate well.

A) B)

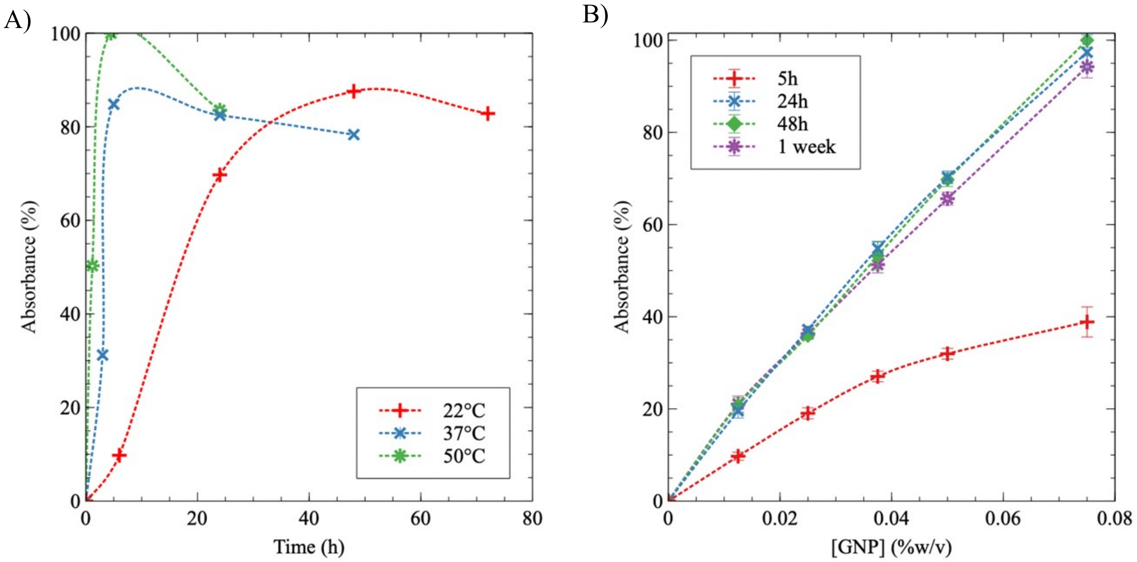

Figure S3. Chitosan (CHI) crosslinking kinetics using genipin (GNP) as monitored by UV-Vis spectroscopy. A) Absorbance as a function of time t and temperature T, at [CHI] = 0.5% w/v and [GNP] = 0.025% w/v; B) Absorbance as a function of time *t* and GNP composition, at [CHI] = 0.5% w/v and 37 °C.

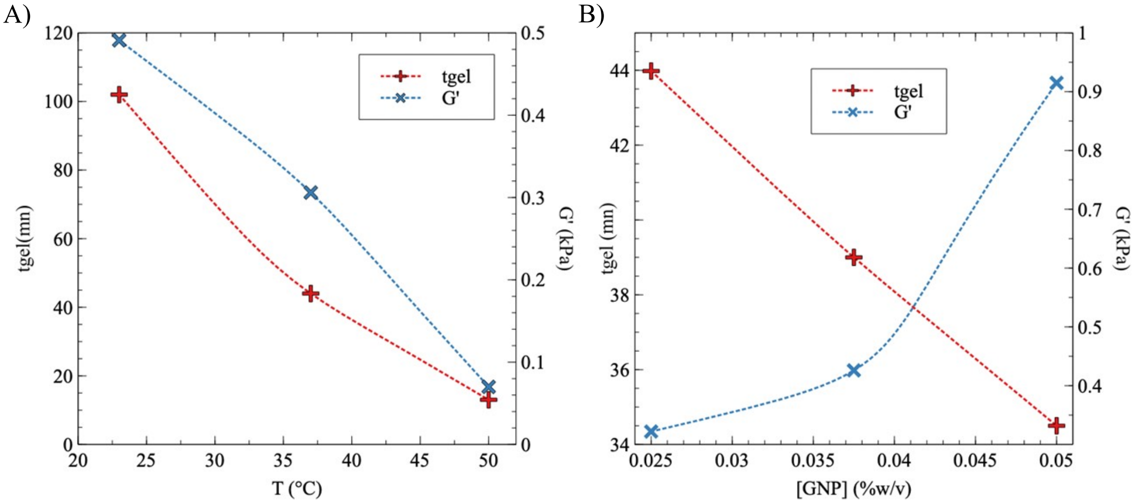

Figure S4. A) Gelation time (t_{gel}), and storage modulus at equilibrium (*G'*) as a function of temperature T, for SA 1% CHI 0. 5% GNP 0.05% w/v solutions; B) Gelation time (t_{gel}) and storage modulus at equilibrium (*G'*) as a function of GNP concentration for SA 1% CHI 0.5% w/v at 37 °C. The decrease in *G'* observed in (A) is due to syneresis.

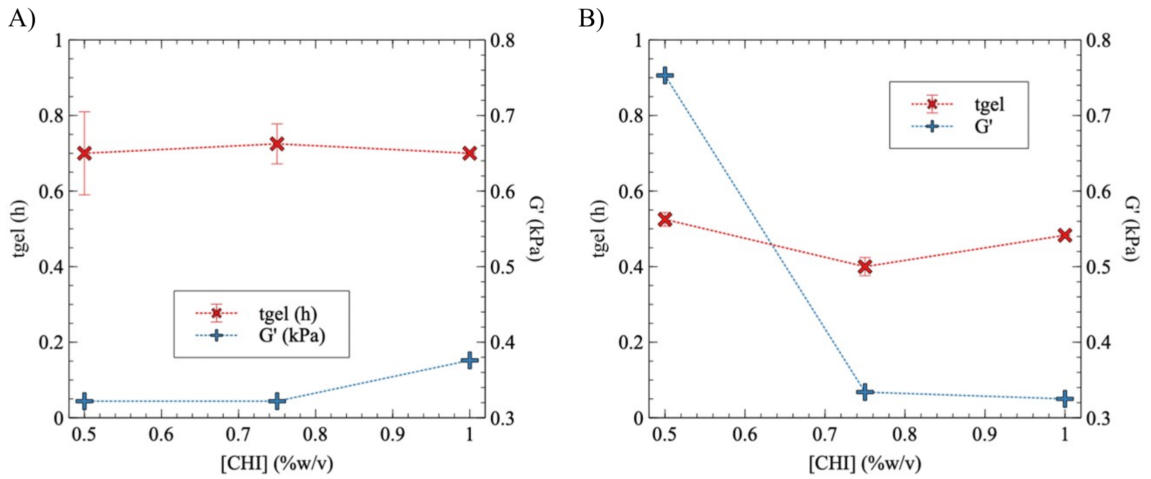

Figure S5. Influence of CHI composition on t_{gel} and final G’ values, at constant SA (1% w/v) content, and at GNP compositions of (A) 0.025% and (B) 0.05% w/v. The decrease in *G’* in (B) is due to syneresis.

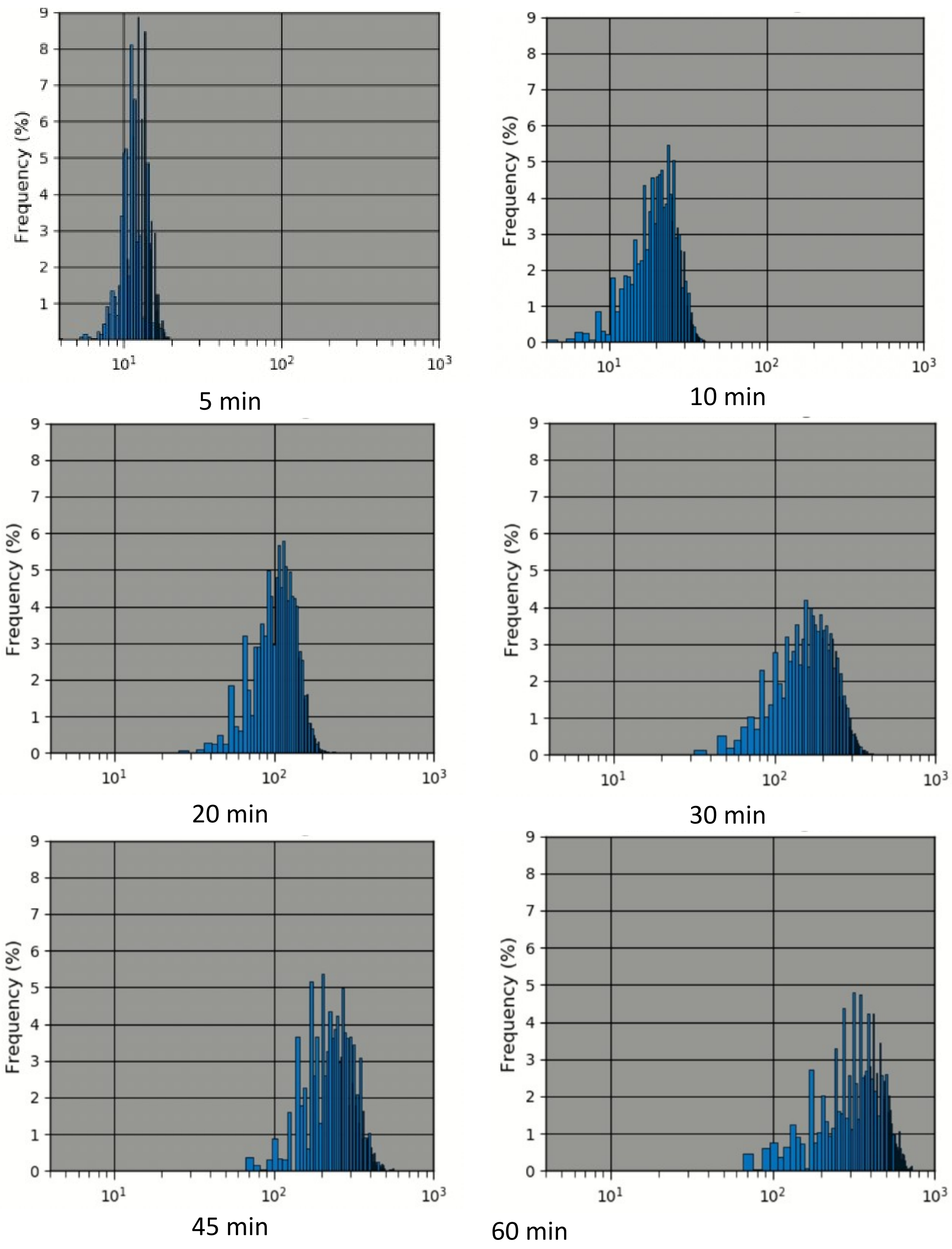

Figure S6. Pore size distributions for different annealing times. The *x*-axis is in μm.

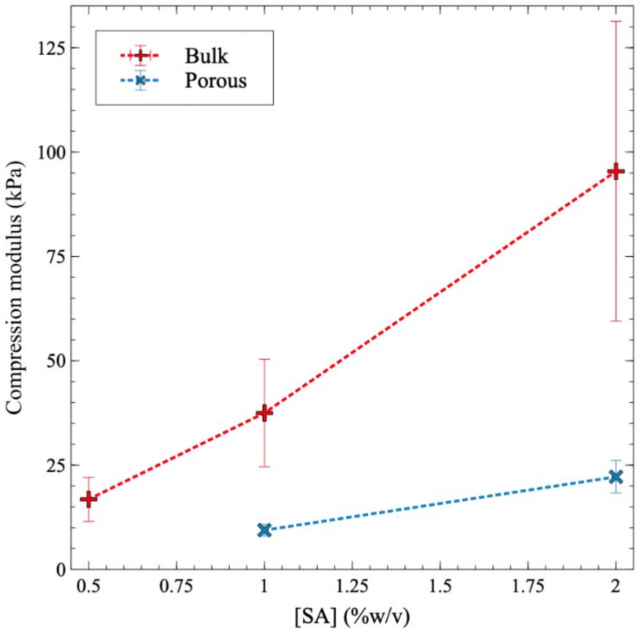

Figure S7. Compression modulus of sodium alginate (SA) bulk and porous (pore size = 180 μm) gels as a function SA composition.

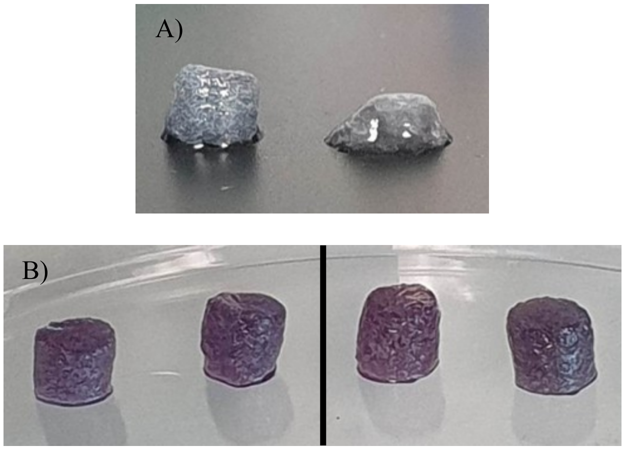

Figure S8. SA 1% CHI 0.75% GNP 0.025% w/v (left) and SA1% CHI0.5% GNP0.025% w/v (right) after (A) 1 week in PBS and (B) 2 weeks in DMEM medium.

A) B)

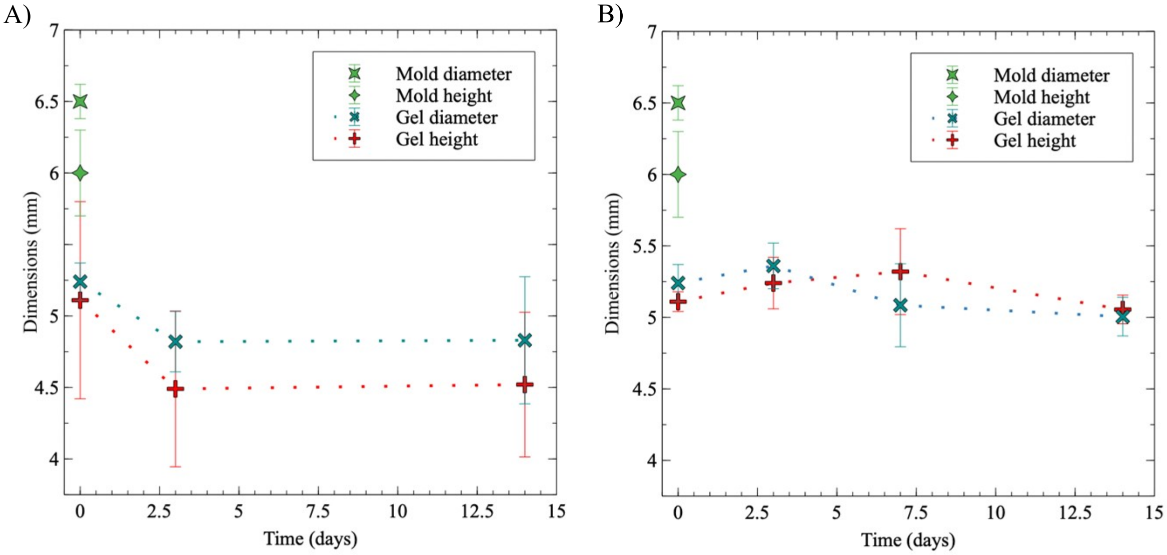

Figure S9. Evolution of porous gels dimensions (1% w/v SA, 0.75% w/v CHI, 0.025% w/v GNP, average pore size = 180 μm) in a) PBS and b) DMEM medium, as a function of time, and compared to the initial PLA molds dimensions.

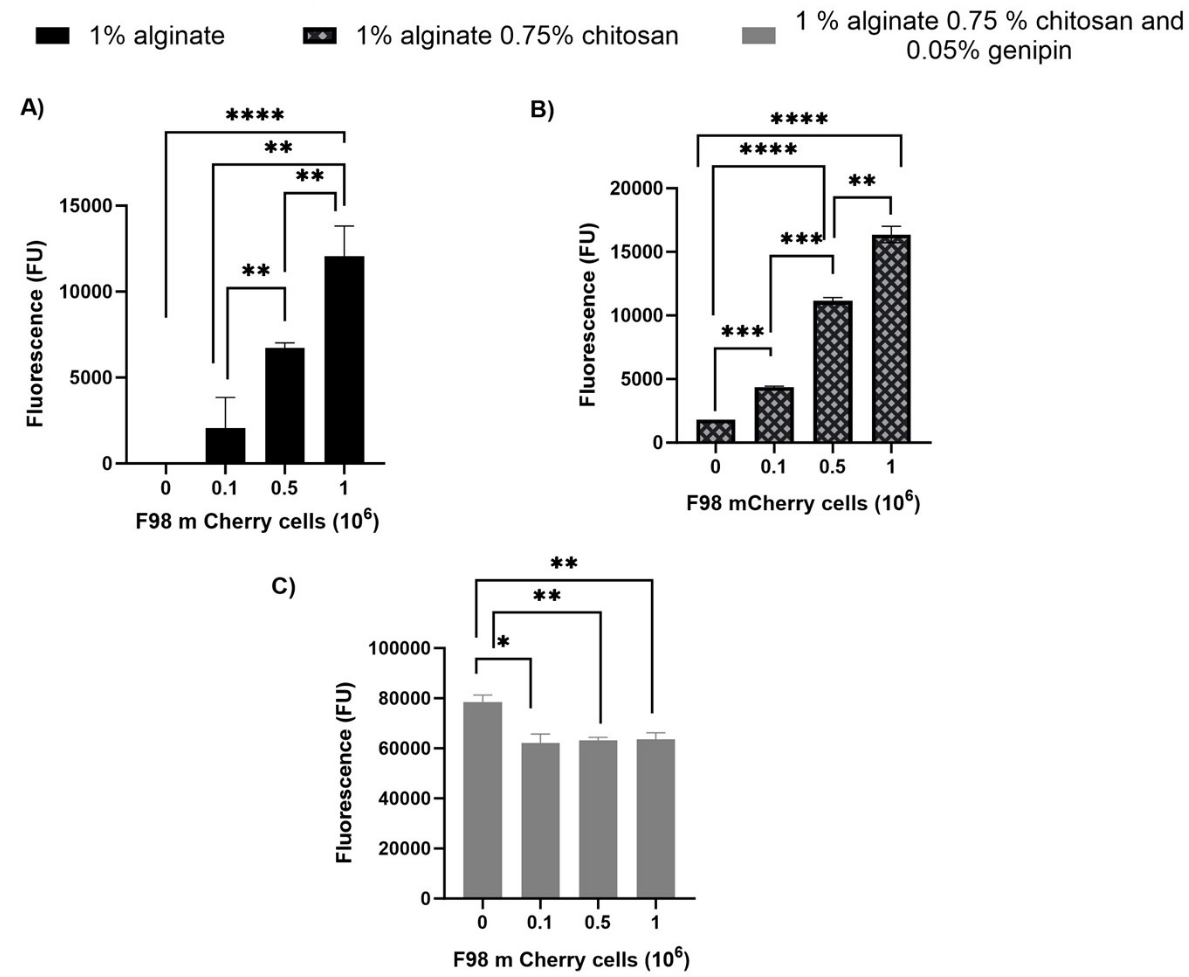

Figure S10. F98 mCherry cells fluorescence intensity in SA 1% hydrogels (A), SA 1% CHI 0.75% hydrogels (B), and SA 1% CHI 0.75% GNP 0.05% w/v hydrogels (C).
